# OX-40 signaling promotes tumorigenesis in CTCL by regulating ERK activation

**DOI:** 10.3389/fimmu.2025.1677140

**Published:** 2025-10-27

**Authors:** Evangelia Papadavid, Fani Karagianni, Eleni-Kyriaki Vetsika, Sara Valero-Díaz, Saire Edith Córdova-Hernández, Christos Daniil, Christina Piperi, Berta Casar

**Affiliations:** ^1^ National Center of Rare Diseases-Cutaneous Lymphoma, Second Department of Dermatology and Venereal Diseases, Attikon University General Hospital, National and Kapodistrian University of Athens, Athens, Greece; ^2^ Centre of New Biotechnologies and Precision Medicine (CNBPM) School of Medicine, National and Kapodistrian University of Athens, Athens, Greece; ^3^ Instituto de Biomedicina y Biotecnología de Cantabria, Consejo Superior de Investigaciones Científicas (CSIC) -Universidad de Cantabria, Santander, Spain; ^4^ Department of Biological Chemistry, National and Kapodistrian University of Athens, Athens, Greece; ^5^ Centro de Investigacion Biomedica en Red de Cancer (CIBERONC), Instituto de Salud Carlos III, Madrid, Spain

**Keywords:** cutaneous T cell lymphoma, CAM assay, mycosis fungoides, Sézary syndrome, OX-40, tumor microenvironment

## Abstract

**Introduction:**

In Cutaneous T-cell Lymphoma (CTCL), T cells can be activated either by cytokines produced by malignant T cells or through immunological synapses, such as the interaction between OX-40 and OX-40L on dendritic cells. Both are co-expressed in tumor cells in Mycosis fungoides/Sézary syndrome and correlate with disease severity markers. Using a model of spontaneous metastasis in chick embryos, the present study aimed to determine the functional role of OX-40 in CTCL and assess its potential as a therapeutic target.

**Methods:**

OX-40 knockout MyLa and SeAx CTCL cells using CRISPR-Cas9 were engrafted onto the chorioallantoic membrane of chick embryos. We assessed tumor growth, dissemination, and TME modulation in the presence or absence of macrophages. Transwell-based transendothelial migration assays and co-culture experiments were performed to further explore the interactions between CTCL cells and macrophages. Angiogenesis and lymphangiogenesis have also been investigated.

**Results:**

OX-40 expression promoted intravasation, metastasis, and cytokine secretion, and increased M2 macrophages. Additionally, it restores transendothelial migration and dissemination in the presence of M2 macrophages, possibly through ERK activation. Co-culture experiments revealed that OX-40 promoted a Th2 cytokine profile in CTCL, correlating with M2 macrophages in xenografts. Although OX-40 did not affect angiogenesis in this model, it promoted lymphangiogenesis via VEGF-C expression.

**Discussion:**

Using the CTCL spontaneous metastasis model in chick embryos, we demonstrated that OX-40 regulates the TME to promote M2 increase, lymphangiogenesis, CAM intravasation, and metastasis. Therefore, the *in vivo* chick embryo metastasis model may serve as a valuable preclinical tool for identifying novel anti-tumor targets in CTCL. The OX-40 axis was identified as a key driver of CTCL progression, promoting tumor growth and metastasis through ERK activation while validating the chick embryo model as a preclinical tool for therapeutic testing.

## 1 Introduction

Cutaneous T-cell lymphomas (CTCL), a group of non-Hodgkin lymphomas marked by the clonal proliferation of malignant T cells in the skin (1, 2) include Mycosis Fungoides (MF) and Sezary syndrome (SS) (1, 3). Both conditions are currently incurable, with advanced stages that often progress to fatal outcomes (4), with a chronic, relapsing course requiring repeated treatment regimens (4). There is a high demand to establish new therapeutic targets for MF/SS that will be effective in the future management of CTCL patients (5, 6). Therefore, a deeper understanding of the key biological mechanisms driving CTCL, particularly in relation to the tumor microenvironment (TME), is important for developing more effective therapeutic schemes and improving patient survival (7).

Among the several emerging immunologic targets, OX-40 (CD134), a type II transmembrane protein belonging to the TNF receptor superfamily, has shown increasing attention. It is expressed on activated CD4+ cells, regulatory T cells (Tregs) and natural killer (NK) cells (8, 9). In recent years, OX-40 has gained interest for its dual role in cancer. Although it is often associated with anti-cancer immunological responses, it has also been shown to promote tumor growth in certain malignancies by modulating key survival pathways, including the MAPK/ERK pathway (10). Malignant T cell-derived cytokines or immunologic synapses with dendritic cells (DCs), such as the OX-40/OX-40L interaction, can activate T cells in CTCL (11). Both OX-40 and OX-40L are co-expressed in tumor cells in MF/SS and have been linked to disease severity markers (12). The proliferation of CTCL cells is suppressed by anti-OX-40 and anti-OX-40L antibodies both *in vitro* and in animal models (12). This suppression was achieved by reducing the phosphorylation of extracellular signal-regulated kinase 1/2 (ERK1/2), Jun N-terminal kinase (JNK), and p38 mitogen-activated protein kinases (p38 MAPK) via the downregulation of a plethora of downstream intracellular signaling axes, such as nuclear factor kappa-light-chain-enhancer of activated B cells (NF-κB), phosphatidylinositol 3-kinase (PI3K)/protein kinase B (AKT), fatty acid synthase (FAS), and B-cell leukemia/lymphoma 2 protein (Bcl-2) (12). These results suggest a potential role for OX-40/OX-40L interactions in fueling MF/SS tumor cell proliferation. Furthermore, the disruption of these interactions could emerge as a novel therapeutic strategy for combating MF/SS.

In the TME, OX-40 expression is frequently upregulated across different immune cell subpopulations, including T cells, DCs, and Tregs, indicating immune activation against tumor cells (8). OX-40 expression on effector T cells indicates their activation status and potential for cytotoxic function against tumor cells, whereas its expression on Tregs increases their suppressive effects on anti-tumor immune responses Additionally, OX-40 expression on DCs enhances their antigen-presenting capabilities, facilitating the priming and activation of tumor-specific T cells. This dual role highlights the complexity of OX40 signaling in modulating immune responses in the TME. Various signaling pathways have been implicated in CTCL; however, the ERK pathway is emerging as a key mediator of tumor cell proliferation, survival, and progression (13). While dysregulated ERK activation in CTCL is associated with oncogenic behaviors, it underscores the need to understand the upstream modulators of this pathway as potential therapeutic targets. We showed that OX-40 influences tumor progression in CTCL, at least in part, through its regulatory effects on ERK activation. Characterization of OX-40 expression patterns in the TME would enable the development of novel immunotherapeutic strategies. Thus, targeting OX-40 signaling may be a potential approach to improve the anti-tumor immune response, leading to better clinical outcomes in patient cohorts. In the present study, we aimed to elucidate the functional significance of OX-40 expression in the *in vivo* spread of MF/SS using a chick embryo metastasis model, and to evaluate its therapeutic potential as an MF/SS target.

## 2 Materials and methods

### 2.1 Cell cultures

The human CTCL, MyLa, SeAx, and HVEC endothelial cell lines were used as previously described (14) and were kindly provided by Dr Michel Laurence (Skin Research Center Service de Dermatologie Hôpital Saint-Louis, INSERM, Paris, France). All cell lines were tested for Mycoplasma using mycoTool Real time PCR (Roche) and authenticated by STR analysis (DNA fingerprinting) for human cell lines by the Promega PowerPlex 18D system and the ThermoFisher Scientific GeneMapper ID-X v1.2 software for analysis of the amplicons. All experiments were performed with cells maintained at ≤ 12 passages to ensure consistency and viability. Use of this human-derived cell line complied with institutional ethical approval by the National Biosafety Commission of Spain, reference A/ES/20/I-85, dated 12/14/21 and the Declaration of Helsinki.

### 2.2 Spontaneous chick embryo metastasis model

The development of the chick embryo chorioallantoic membrane (CAM) model has been described previously (15, 16). Briefly, the eggs were first prepared for xenografting tumor cells, and then tumor cells were prepared for grafting onto the CAM. Upon grafting, the tumor and chick embryo tissues were harvested as previously described (14). The chick embryo is not considered a living animal until day 17 of development; therefore, the chick CAM assay did not require administrative procedures for approval by the Ethics Committee for Animal Experimentation. This approach does not require permission to experiment on animals because the CAM was not innervated, and studies were stopped before the development of brain regions linked to pain perception. Every experiment was carried out in compliance with the national animal care criteria stipulated in the EU Directive.

### 2.3 Quantitative real-time PCR

Extraction of genomic DNA, detection of human cells in chick tissues, and quantification were performed as previously described (14). Genomic DNA was extracted from the harvested tissues using the Qiagen DNA purification system (#158906; #158910; #158914; Qiagen, Hilden, Germany). To detect human cells in the chick tissues, primers specific for the human Alu sequences (sense: 5’ ACGCCTGTAATCCCAGGACTT; 30antisense: 50 TCGCCCAGGCTGGCTGGGTGCA 3’) were used to amplify the human Alu repeats present in genomic DNA that was extracted from chick tissues. The real-time PCR used to amplify and detect Alu sequences contained 30 ng of genomic DNA, 2 mm MgCl_2_, 0.4 µm each primer, 200 µm DNTP, 0.4 units of Platinum Taq polymerase (Invitrogen Corporation, Carlsbad, CA, USA), and a 1:100,000 dilution of SYBR green dye (Molecular Probes, Eugene, OR, USA). Each PCR was performed in a final volume of 10 µL under 10 µL of mineral oil with the iCycler iQ (Bio-Rad laboratories, Hercules, CA, USA) under the following conditions: polymerase activation—95 °C for 2 min, 40 cycles at 95 °C for 30 s, 63 °C for 30 s, 72 °C for 30 s. A quantitative measure of amplifiable chick DNA was obtained through the amplification of the chick GAPDH genomic DNA sequence with chGAPDH primers (sense: 5’ GAGGAAAGGTCGCCTGGTGGATCG 3’; antisense: 5’ GGTGAGGACAAGCAGTGAGGA ACG 3’) using the same PCR conditions as described for Alu. The fluorescence emitted by the reporter dye was detected online in real-time, and the threshold cycle (Ct) of each sample was recorded as a quantitative measure of the amount of PCR product in the sample. The Ct is the fractional cycle number at which the fluorescence generated by the reporter dye exceeds a fixed level above baseline. When indicated, the Alu signal was normalized against the relative quantity of GAPDH and expressed as ΔCt = (Ct_GAPDH_ − Ct_Alu_). The changes in Alu signal relative to the total amount of genomic DNA (and, hence, changes in the quantity of human DNA in the chick tissue) were expressed as ΔΔCT = ΔCt_control_ − ΔCt_treatment_. Relative changes in metastasis were then calculated as 2ΔΔCT. Each assay included negative control, positive control, no-template control, and experimental samples in triplicate. To approximate the actual number of tumor cells, present in each tissue sample, a standard curve was generated through quantitative amplification of genomic DNA extracted from a serial dilution of MyLa and SeAx cells, respectively, mixed with individual chick lung homogenates. By interpolating the Alu signal from experimental samples with the standard curve, the actual number of tumor cells/lung could be determined over a range of 50–100,000 cells/lung.

### 2.4 Selection of CTCL cell dissemination variants using the chick embryo model

Excision of primary tumors from the CAM under sterile conditions took place; they were washed in SF-DMEM (Sigma Aldrich, Merck), minced and incubated for 4h in the presence of digestive enzymes [1 mg/ml dispase (Sigma Aldrich, Merck); 1 mg/ml collagenase (Sigma Aldrich, Merck)] in SF-DMEM. Following mechanical disruption using 100 μm cell strainers, the dissociated tissue was rinsed with 10% FBS/DMEM, and the resultant cell suspensions from individual tumors were pooled and seeded for propagation *in vitro* in 10% FBS/DMEM. After 2–3 weeks, the expanded tumor cells were grafted onto new CAMs for another round of tumor growth and cell isolation. This process of tumor cell grafting, followed by tumor cell isolation, was repeated six times, yielding a series of cell lines. The MyLa and SeAx sublines were then transplanted into the CAM of day 10 chick embryos for tumor development. After 7 days, portions of CAM distal from the primary tumor site were harvested under sterile conditions and dissociated by dispase treatment and mechanical disruption. Within two-three weeks of the dissociated cells being plated for *in vitro* propagation, visible tumor cell outgrowths were identified by their cobblestone-like appearance inside a layer of chick embryonic fibroblasts. A highly disseminating subline of MyLa and SeAx (hi/diss) was produced by isolating and pooling the tumor cell colonies.

### 2.5 Western blot

Radioimmunoprecipitation Assay buffer (RIPA, Sigma Aldrich) supplemented with phosphatase and protease inhibitors (Roche, Germany) was used to lyse the microtumors. Following normal western blotting, whole-cell lysates were subjected to acrylamide SDS-PAGE and then deposited onto a nitrocellulose blotting membrane (Amersham Protran, GE Healthcare Life Science). The primary and secondary antibodies were diluted to 1:1000 and 1:5000, respectively. The following primary antibodies were used: phosphor-extracellular signal-regulated kinase phosphor (*p*-ERK; #ab627545; Santa Cruz), p44/42 mitogen-activated protein kinase (MAPK; ERK1/2; clone 137F5; # ab390779; Cell Signaling Technology), phospho ELK-1 thr417 (# PA5-36642; Invitrogen), ELK-1 (# MA5-15311; Invitrogen), phospho p90-RSKT359/S362 (# SAB5700397; Sigma Aldrich, MERCK), Rsk-1 [(10B1D7); # sc-81162; Santa Cruz Biotechnology], vascular endothelial growth factor-C (VEGF-C) (Abcam), Glyceraldehyde 3-phosphate dehydrogenase (GAPDH; 6C5; Abcam), used as a loading control, and α-tubulin (Sigma-Aldrich). The following secondary antibodies were used: P/N 925-32212 (#ab2716622), P/N 925-32213 (#ab2715510) and P/N 926-32213 (#ab621848). Signal detection and imaging were performed using a ChemiDoc MP Imaging System (Bio-Rad).

### 2.6 OX-40 CRISPR-cas9 knockout

To knock out OX-40 in CTCL cell lines, OX-40 CRISPR/cas9 edits were generated using a protocol previously described by Santa Cruz Biotechnology (17, 18). In a 6-well tissue culture plate, 1.5 x 10^5^–2.5 × 10^5^ CTCL cells were seeded in 3 ml of antibiotic-free RPMI-1640 (Gibco, Thermo Scientific) per well, 24h prior to transfection with 1-3 μg DNA per well using Ultracruz Transfection Reagent. Six hours post-transfection, the medium was replaced with fresh medium containing 5 μg/ml puromycin (Stem Cell Technologies). The cells were selected for a minimum of 3-5 days. Freshly prepared selective media containing 5 μg/ml puromycin were used to replace the medium roughly every two–three days. To verify full allelic knockouts, single-cell colonies underwent comprehensive phenotypic and/or genotypic characterization using western blotting and real-time (RT)-PCR.

### 2.7 Overexpression of OX-40

The human OX40 full-length Gene in Lentivector, Endotoxin-free DNA was obtained from G&P Biosciences (SKU#: LTP0103). The Lentiviral expression vector pLTC, which can be utilized for both temporary and stable expression in mammalian cells, was created by subcloning the full-length human OX40/CD134 gene with an upstream CMV promoter. To produce high-titer lentiviral particles, the plasmid was co-transfected into HEK293 cells along with LentiPAK DNA mix (SKU# LP-001). The mock control plasmid was empty. After 48 h, the viral supernatants were collected and used to inoculate MyLa and SeAx cells. Following infection, cells were cultured cells for two weeks in a medium containing 1 μg/ml of blasticidin (Sigma Aldrich), and western blot analysis confirmed the expression of OX-40.

### 2.8 Transendothelial cell migration using transwell-based transendothelial migration assay

HVEC endothelial cells (1x10^5^) were cultivated to confluence on the insert undersides of 6.5 mm-Transwells with 8μm pores (Corning Inc., MA) in 0.1 ml endothelial culture media. Individual inserts were filled with 5x10^4^ cells in 0.1 ml). FBS/DMEM (5%) was then added to the lower chamber. Fluorescence microscopy was used to image and quantify the transmigrated cells after 24 h, and the proportion of green-fluorescent cells to all cells was calculated.

### 2.9 Isolation and culture of M1 and M2 macrophages

CD14^+^ monocytes were extracted as previously reported (19). Briefly, the macrophage culture media contained RPMI (Thermo Fisher), 10% FBS (Thermo Fisher), 0.5% P/S (Thermo Fisher), 10 mM HEPES (Thermo Fisher), 0.1% 2-Mercaptoethanol (ThermoFisher), and recombinant human GM-CSF at 20 ng/ml (Peprotech). Freshly harvested primary monocytes were plated at a density of 20–25 M per 10 cm plate. Additional macrophage medium was introduced the next day (day 1), and on day 4, the macrophage medium was changed. Macrophages differentiated into M1 or M2 subtypes on day 7. Prior to the M1-like macrophage tests, macrophage media were supplemented with IFN-γ (50 ng/ml; Peprotech) and lipopolysaccharide (LPS; 10 ng/ml; eBioscience) for 72h. IL-4 (20 ng/ml; Peprotech) and IL-13 (20 ng/ml; Peprotech) were administered to the macrophage media for 72h before testing for M2-like macrophages. Both M1-like and M2-like macrophage media were devoid of cytokines after they were plated for studies (macrophage culture media + GM-CSF alone) to prevent activation of cytokines from affecting cancer cells. In the transendothelial migration model, M1 or M2 macrophages were incubated for 24h, and in the chick embryo spontaneous metastasis model, for seven days. Conditioned media (CM) generated after macrophage differentiation were collected and referred to as M1 or M2 differentiation CM. Following differentiation, macrophages were washed twice with PBS and cultured for an additional 48 h in RPMI supplemented with 5% FCS (without IFN-γ or IL-4/IL-13). The resulting CM were harvested and designated as M1 CM and M2 CM, respectively. Media were centrifuged to remove cellular debris and stored in aliquots at −80 °C until use. M1 CM and M2 CM (200 µl) were subsequently added to each CAM xenografted tumor for 7 consecutive days.

### 2.10 Immunofluorescence staining of frozen tumor sections

Tumors were excised, embedded in optimal cutting temperature compound (OCT; Thermo Fisher Scientific), and snap-frozen in isopentane cooled with liquid nitrogen until the OCT had set. Samples were stored at −80 °C until use. Sections (20 µm) were cut at −18 °C using a cryostat and mounted onto glass microscope slides. Tissue sections were fixed by immersion in 3% formaldehyde for 30 min, followed by rehydration in PBS. Permeabilization was performed with 1% BSA and 0.1% Triton X-100 in PBS for 30 min. Slides were then blocked with 5% BSA in PBS for 1 h at room temperature. Primary antibodies against OX40 (CD134/OX-40; #ab270727; Abcam), CD163 (#ab87099; Abcam), and CD86 (#ab220188; Abcam) (1:100 dilution in 5% BSA) were applied (20 µl per section), and slides were incubated overnight at 4 °C in a dark, humidified chamber. The following day, sections were washed three times with PBS containing 0.05% Tween-20 and incubated with Alexa Fluor 405, FITC, or Alexa Fluor 594-conjugated secondary antibodies (1:200 dilution in 5% BSA; 20 µl per section; Life Technologies) for 1 h at room temperature in the dark. After three additional PBS washes, slides were mounted with EverBrite™ Hardset Mounting Medium (Cambridge Bioscience) and coverslipped. Images were acquired using a Nikon confocal microscope.

### 2.11 Immunofluorescence for angiogenesis and lymphangiogenesis

The effects of OX-40 on tumor cell-induced angiogenesis and lymphangiogenesis were evaluated using a collagen on plant angiogenesis test, as previously described (20). In short, MyLa and SeAx cells were introduced at a density of 10^6^ cells/ml to 2.2 mg/ml neutralized native type I collagen (BD Biosciences), and 30 µl-droplets of the mixture were then polymerized across grid meshes (3x4 mm). This process created three-dimensional (3D) images of plants, which were then incubated for 30 min at 37 °C. On the CAM of 10-day-old chick embryos that were cultured *ex ovo*, solidified onplants were grafted (four onplants/embryo). Using a stereoscope, newly formed blood vessels that had expanded into three-dimensional collagen rafts were scored across the lower mesh in less than 2 h, and the number of grids with newly created blood vessels was divided by the total number of scored grids to determine the angiogenic index (number of bold vessels/square). The allantoic vein of onplant-bearing embryos was injected with 200 μg of Rhodamine-conjugated Lens culinaris agglutinin (LCA; Vector labs) in 0.2 ml PBS in order to observe the angiogenic blood vessels within collagen rafts. Sections of CAM containing onplants were examined for fluorescence using a digital video camera attached to an Olympus microscope. To examine lymphangiogenesis, immunofluorescence tests were performed using goat anti-rabbit IgG (H+L) secondary antibodies, FITC, and anti-Prox-1 (Abcam and Invitrogen). The primary antibody was washed and incubated for two hours after the parts of the CAM were rinsed in PBS-0.05% Tween 20 (Sigma-Aldrich). Next, secondary antibodies that had been conjugated to FITC were incubated for one hour. FITC and Lectin Red were excited at wavelengths of 488 and 543 nm, respectively, using an Olympus microscope. The images were subsequently processed and examined using FIJI Image J (21), an open-source platform based on ImageJ.

### 2.12 Cytokine measurement

With a few adjustments, the tissue supernatant was prepared for cytokine testing, as previously described (22). Briefly, 0.5 ml of RPMI 1640 (per 100 mg tissue) was added to the CTCL tumor tissues after removal and chopped into tiny pieces. The tissue fragments were centrifuged at 1500 rpm. After being moved to a 1 ml syringe, the supernatant was filtered using 0.22 μm and 10 μm cell strainer.

### 2.13 Angiogenesis *ex ovo* chick embryo CAM assay

As previously mentioned, CAM angiogenesis experiments were performed (20). Briefly, type I rat tail collagen (BD Biosciences) was produced and neutralized at a final concentration of 2 mg/ml. Cells were added to the collagen mixture at a final concentration of 0.5×10^6^ cells/ml of collagen solution. Twenty microliters of the final collagen mixture were polymerized between the two nylon-gridded meshes to form an onplant. Four to six embryos (three to five embryos per variable) were placed on the CAMs of shell-less day 10 embryos growing *ex ovo*. After 72 h, angiogenic vessels were scored above the upper mesh and an angiogenic index (number of grids with newly generated blood vessels over the total number of grids scored) was calculated for each plant. A minimum of two runs were conducted for each experiment.

### 2.14 CD86, CD163 and VEGF-C expression

SuperScript II RNase H-reverse (Invitrogen CA) was used to synthesize first-strand DNA from one μg total RNA. Human VEGF-C (accession No. NM005429:5′-CTGCTCGCCGCTGCGCTG and 5′-GTGCTGGTGTTCATGCACTGCAG), human CD86 (accession No.NM175862: 5′- TGCTCATCTATACACGGTTACCand 5′- TGCATAACACCATCATACTCGA, human CD163 (accession No.NM004244:5′- ATCAACCCTGCATCTTTAGACA and 5′- CTTGTTGTC ACATGTGATCCAG and human GAPDH (accession numbers. BC004109:5′-CGGAGTCAACGGATTTGGTCGTAT and 5′-AGCCTTCTCCATGGTGGTGAAGAC) were used as forward and reverse primers, respectively, for polymerase chain reaction (PCR). To obtain a single product with an appropriate base pair size within the linear range of the reaction, all PCR parameters and primers were adjusted. The ratio of target messenger RNA (mRNA) to GAPDH mRNA for each sample was used to determine the target mRNA expression level.

### 2.15 Statistical analysis

Data processing and statistical analyses were conducted using GraphPad Prism 9 (GraphPad Software Inc., San Diego, CA, USA) and Microsoft Excel 16.3 (Microsoft Corporation, Redmond, WA, USA). Normal distribution of the samples was evaluated using the D’Agostino-Pearson test with an alpha value of 0.05. Unpaired t-tests with Welch’s correction, Mann-Whitney U tests, or Brown-Forsythe and Welch ANOVA tests with Dunnett’s T3 multiple comparison test were used for comparisons. The associated Figure legend for each experiment indicates the number of independent experiments and the sample size. The experimental values of the graphs are presented as the mean ± SEM. For two-sided tests, differences were considered statistically significant at p<0.05.

## 3 Results

### 3.1 OX-40 expression promotes high-disseminated CTCL cell intravasation and colonization to distal organs

**Figure 1:**
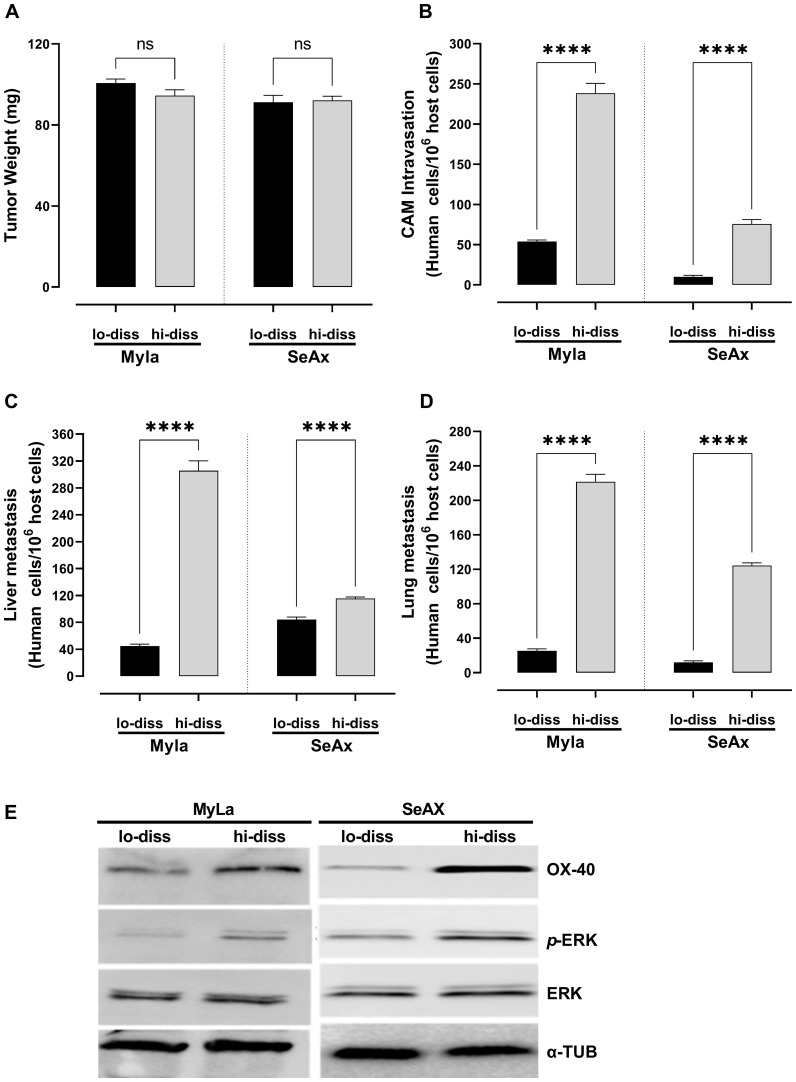
OX-40 expression promotes CTCL cell intravasation and colonization to distal organs. Ability of high- and low-disseminated OX-40-expressing MyLa and SeAx to affect **(A)** tumor weight (unpaired t-test with Welch’s correction); **(B)** CAM intravasation (Mann-Whitney U test); and distal metastasis (unpaired t-test with Welch’s correction) to **(C)** liver; and **(D)** lung. **(E)** Western Blot analysis of high- and low-disseminated cells to detect OX-40, *p*-ERK, and ERK (unpaired t-test with Welch’s correction). Data are presented as mean ± SEM of three independent experiments. MyLa: 18 embryos n=3 independent experiments; SeAx: 15-18 embryos, n=3 independent experiments; ****p < 0.0001; ns, not significant Hi-diss, highly disseminated; Lo-diss, low-disseminated; CAM, chorioallantoic membrane; ERK, extracellular signal-regulated kinase; tub, tubulin.

The metastatic potential of disseminated CTCL cells was investigated by dichotomizing them into high- and low-dissemination variants based on their invasion ability using the chick embryo CAM model to assess their ability to enter circulation and colonize distant organs. Subsequently, their effects on tumor weight, intravasation into the CAM, and distal metastasis were assessed. Tumor weight did not differ significantly between the low- and high-disseminated CTCL cells (**Figure 1A**). The highly disseminated CTCL cells exhibited a significantly greater ability to intravasate through the vascular endothelium (75.7% MyLa, p<0.0001; 92.2% SeAx, p<0.0001; **Figure 1B**) and metastasis to the liver (87.5% MyLa, p<0.0001; 49.7% SeAx, p<0.0001; **Figure 1C**) and lungs (82.8% MyLa, p<0.0001; 85.5% SeAx, p<0.0001; **Figure 1D**) than the low-disseminated cells. Notably, Western blot analysis of highly disseminated cells revealed significant upregulation of OX-40 expression as well as increased ERK1/2 phosphorylation (**Figure 1E**, **Supplementary Figures S1, S3**), suggesting that the OX-40/ERK signaling axis is essential for promoting the metastatic potential of these cells. In contrast, low-disseminated cells exhibited significantly lower OX-40 levels (52% MyLa, p<0.005; SeAx 65%, p<0.005) and reduced ERK1/2 activation (61% MyLa, p<0.005; 51% SeAx, p<0.01), indicating a correlation between OX-40 expression, ERK activation, and the extent of tumor cell dissemination. These findings imply that OX-40 stimulates the ERK signaling pathway, which is important for intravasation and subsequent migration of tumor cells to distant regions, thus boosting CTCL metastasis. This also highlights the critical role of OX-40 in facilitating metastasis and entry of tumor cells into the vasculature.

### 3.2 OX-40 loss reduces tumor weight, transendothelial migration, CAM intravasation and distal metastasis via ERK1/2 pathway

**Figure 2:**
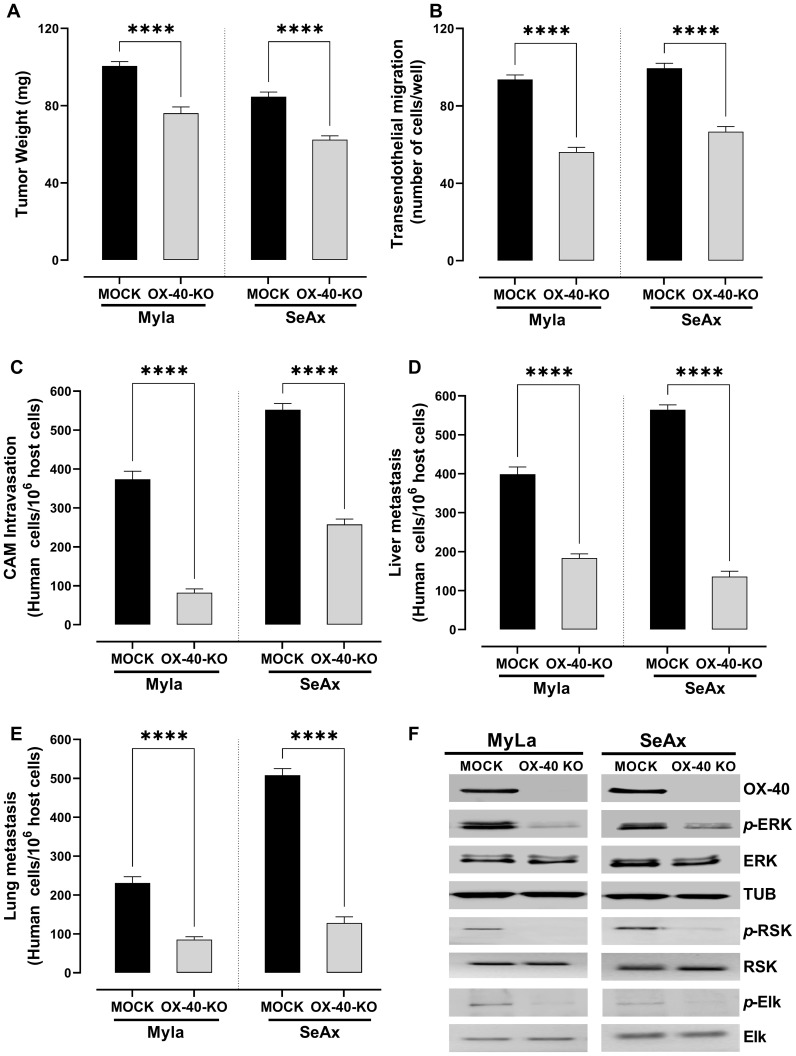
Loss of OX-40 reduces tumor weight, transendothelial migration, CAM intravasation, and distal metastasis through inhibition of the ERK1/2 signaling pathway. OX-40 KO MyLa and SeAx cells engrafted in the chick embryo CAM model significantly reduced **(A)** tumor weight (unpaired t-test with Welch’s correction) **(B)** transendothelial migration (unpaired t-test with Welch’s correction) **(C)** CAM intravasation (Mann-Whitney U test) **(D, E)** distal metastasis, and **(F)** and reduced phosphorylation of ERK1/2, ELK and RSK in CAM-grafted CTCL tumors, as shown by Western blot analysis (unpaired t-test with Welch’s correction). Data are presented as mean ± SEM of three independent experiments. MyLa: 11-12 embryos, n=3 independent experiments; SeAx: 16 embryos, n=3 independent experiments; ****p < 0.0001; ns, not significant [unpaired t-test with Welch’s correction or Mann-Whitney U test (tumor weight)]. KO, knockout; CAM, chorioallantoic membrane; ERK, extracellular signal-regulated kinase; tub, tubulin.

To directly investigate the functional role of OX-40 in CTCL progression, we utilized CRISPR/cas9 technology to generate OX-40 knockout CTCL cell lines. OX-40 knockout CTCL cells engrafted into the chick embryo model resulted in a significant reduction in tumor weight (28% in MyLa embryos, p < 0.0001; 40% in SeAx embryos, p < 0.0001; **Figure 2A**) compared with mock CTCL cells, indicating the critical role of OX-40 in tumor progression. Additionally, the loss of OX-40 in CTCL cells reduced transendothelial migration (**Figure 2B**), which underscores the critical role of OX-40 in CTCL cell migration across endothelial barriers and reveals its potential as a therapeutic target in controlling disease progression. Significant inhibition was also observed in CAM intravasation (72.4% in MyLa embryos, p <0.0001; 55.3% in SeAx embryos, p <0.0001; **Figure 2C**) and metastasis to the liver (60.7% in MyLa embryos, p <0.0001; 73.2% in SeAx embryos, p <0.0001; **Figure 2D**) and lung (69.4% in MyLa embryos, p <0.0001; 70.1% in SeAx embryos, p <0.0001; **Figure 2E**) compared to the controls, highlighting the critical role of OX-40 in driving tumor metastasis, suggesting its potential as a therapeutic target for limiting CTCL dissemination. One of the key pathways activated by OX-40 signaling has been suggested to be the MAPK/ERK. In agreement with this, a reduction in ERK1/2 phosphorylation (79% MyLa p<0.005; SeAx 53%, p<0.005) was observed in OX-40 knockout CTCL cells compared to controls by western blot analysis (**Figure 2F**; **Supplementary Figure S2**). We further examined whether OX-40 activation affects downstream ERK signaling. Our results showed that OX-40 stimulation increased both the phosphorylation of ERK as well as its downstream substrates, RSK and ELK (**Figure 2F**, **Supplementary Figure S5**). These findings indicate that OX-40 activates the ERK pathway at multiple levels, engaging not only ERK itself but also downstream effectors that drive cellular processes such as invasion, migration, proliferation, and survival.

### 3.3 OX-40 modulates transendothelial migration and metastasis in CTCL via M2 macrophage-dependent mechanism

**Figure 3:**
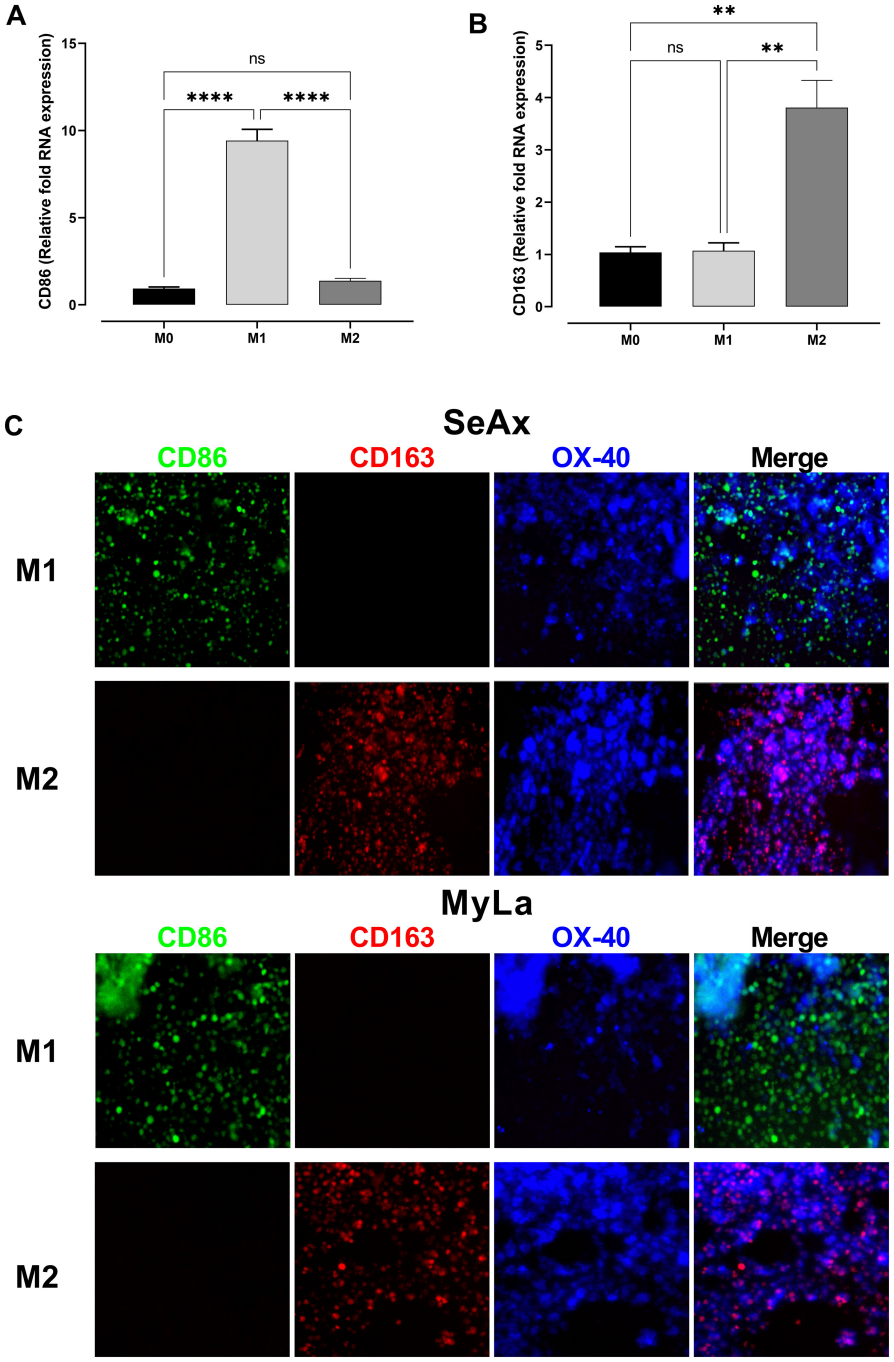
Confirmation of macrophage polarization and the involvement of M2 macrophages in OX-40 KO tumor development. Comparative RNA levels of polarization markers in monocytes: CD86 M1 **(A)** and CD163 M2 **(B)**. Immunofluorescence stain on frozen tumor sections of SeAx/MyLa xenografts demonstrating the existence of the CD86 + (M1) and CD163 + (M2) macrophages in the tumor microenvironment **(C)**. Data are presented as mean ± SEM of three independent experiments. MyLa: 8-12 embryos, n=3 independent experiments; SeAx: 16 embryos, n=3 independent experiments; ****p < 0.0001; **p<0.01; ns, not significant.

We conducted RNA quantification tests in M1- and M2-polarized monocytes to confirm macrophage polarization. We evaluated the expression of two distinctive markers: CD163 for M2 macrophages, and CD86 for M1 macrophages (**Figures 3A, B**). The successful polarization of monocytes into the corresponding M1 and M2 phenotypes was validated by the differential expression patterns. Furthermore, we confirmed the presence of CD86^+^ (M1) and CD163^+^ (M2) macrophages in the tumor microenvironment by using immunofluorescence analysis on frozen tumor sections derived from SeAx/MyLa cell xenografts in the CAM of chicken embryos (**Figure 3C**). To clarify whether the effect of M2 macrophages on the metastatic behavior of OX-40 knockout cells is mediated by soluble factors or requires direct cell–cell contact, we conducted conditioned medium (CM) experiments (**Supplementary Figure S4**). The results demonstrated that CM derived from M2 macrophages was not sufficient to restore the metastatic phenotype. This indicates that direct physical interactions are essential for the pro-tumoral effect.

**Figure 4:**
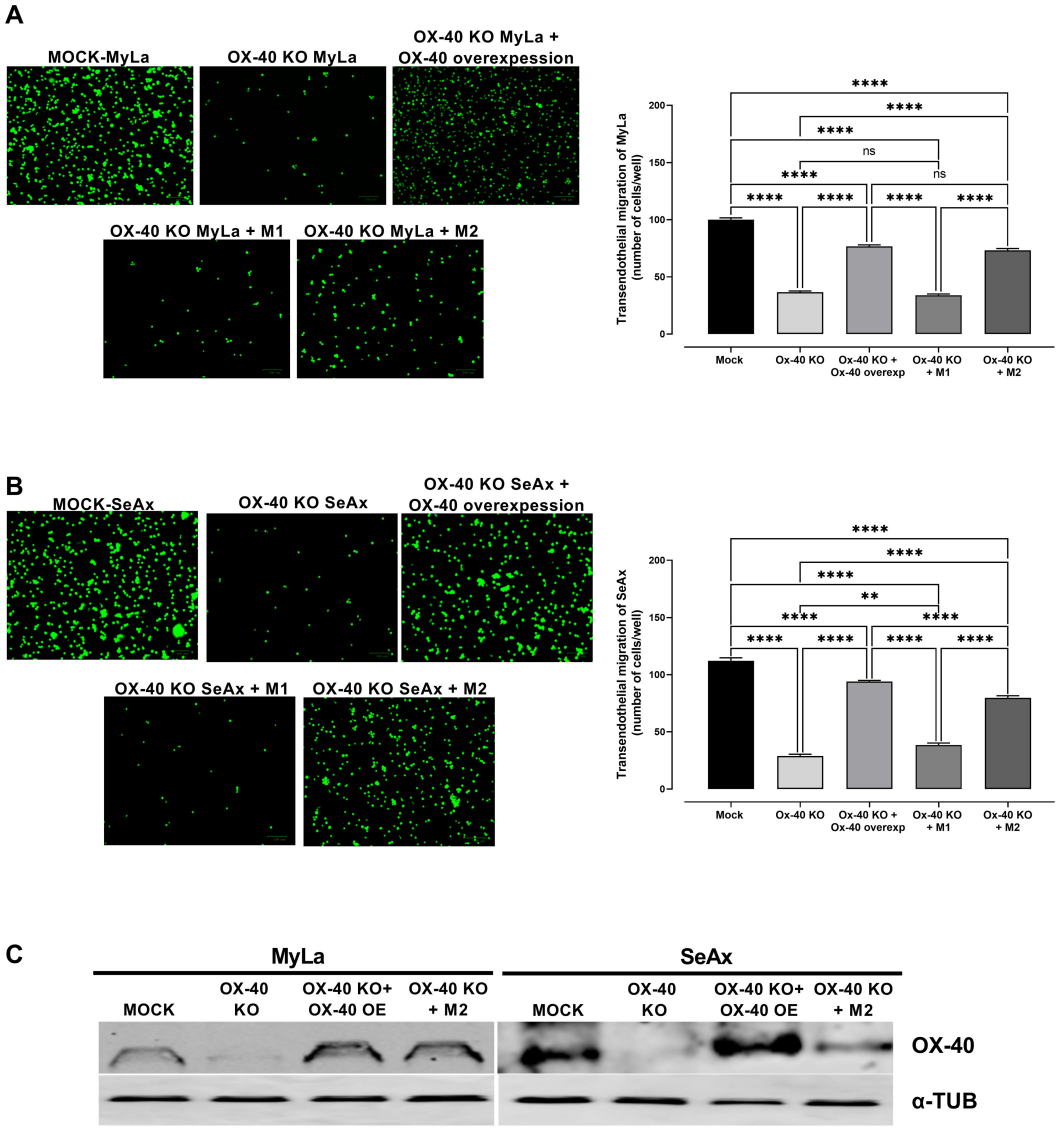
The effect of loss of OX-40 on MyLa cells and SeAx in transendothelial migration in the presence or absence of macrophages. Representative fluorescence images of **(A)** co-cultures of OX-40 KO MyLa cells with HVEC endothelial cells and M1 and M2 macrophages **(B)** co-cultures of OX-40 KO SeAx cells with HVEC endothelial cells and M1 and M2 macrophages **(C)** Western Blot analysis of co-cultures for the detection of OX-40. Data are presented as mean ± SEM of three independent experiments. MyLa: 15 embryos n=3 independent experiments; SeAx: 16 embryos, n=3 independent experiments; **p < 0.01, ****p < 0.0001; ns: not significant (Brown-Forsythe and Welch ANOVA tests with Dunnett’s T3 multiple comparison test). KO, knockout; OE, overexpression; M, macrophages.

**Figure 5:**
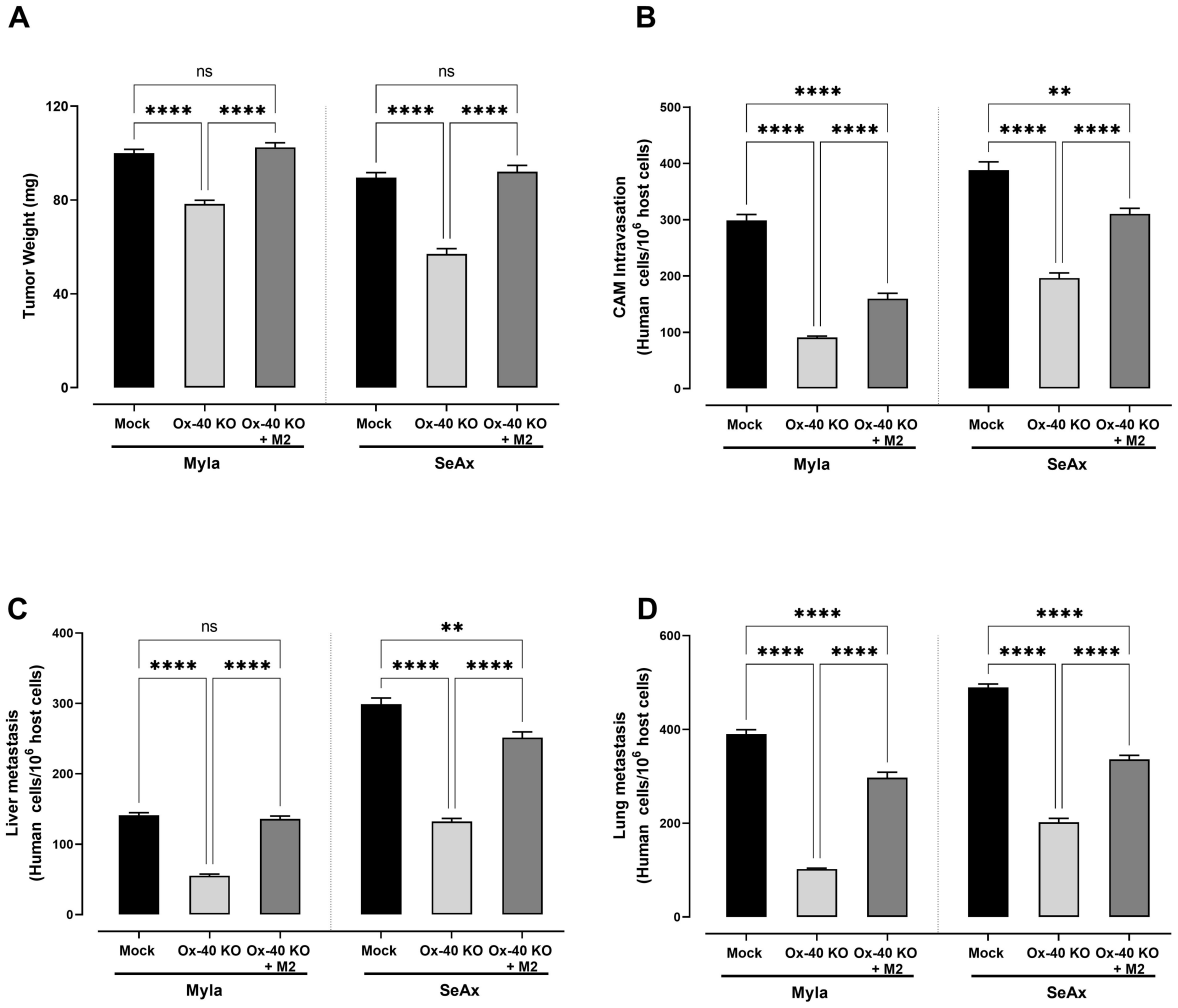
The effect of loss of OX-40 on CTCL cells in tumor weight, CAM intravasation and distal metastasis in the presence and/or absence of macrophages. OX-40 KO CTCL cells significantly reduced **(A)** tumor weight (unpaired t-test with Welch’s correction) **(B)** transendothelial migration (unpaired t-test with Welch’s correction) **(C)** CAM intravasation (Mann-Whitney U test) **(D)** distal metastasis and these effect were restored in the presence of M2 macrophages. Data are presented as mean ± SEM of three independent experiments. MyLa: 15-16 embryos n=3 independent experiments; SeAx: 16 embryos, n=3 independent experiments; **p < 0.01, ****p<0.0001; ns: not significant (Brown-Forsythe and Welch ANOVA tests with Dunnett’s T3 multiple comparison test). KO, knockout; OE, overexpression; M, macrophages.

To further explore the role of OX-40 in the TME of CTCL, we co-cultured CTCL cells and OX-40 knockout CTCL cells with macrophages (M1 or M2) and evaluated their transendothelial migration ability. Interestingly, when OX-40 knockout CTCL cells were co-cultured with M2 macrophages, their transendothelial migration ability was significantly increased compared to that of OX-40 KO CTCL cells (**Figures 4A-C**). Moreover, when OX-40 was overexpressed in OX-40 knockout CTCL cells, the inhibition was elevated but did not reach baseline levels compared to CTCL cells (**Figures 4A-C**). When M1 macrophages and OX-40 knockout CTCL cells were co-cultured, no statistically significant change was observed compared to that in OX-40 KO CTCL cells. Moreover, the reduction in tumor weight (26.2% MyLa, p <0.0001; 42.1% SeAx, p <0.0001; **Figure 5A**), CAM intravasation (72.9% MyLa, p <0.0001; 57.7% SeAx, p <0.0001; **Figure 5B**), and distal metastasis to the liver (77.2% MyLa, p <0.0001; 61.5% SeAx, p <0.0001; **Figure 5C**) and lung (80.5% MyLa, p <0.0001; 54.7% SeAx, p <0.0001; **Figure 5D**) increased in the presence of M2-like macrophages.

### 3.4 M2 macrophages restore IL-5 and IL-13 expression in OX-40 knockout CTCL cells

**Figure 6:**
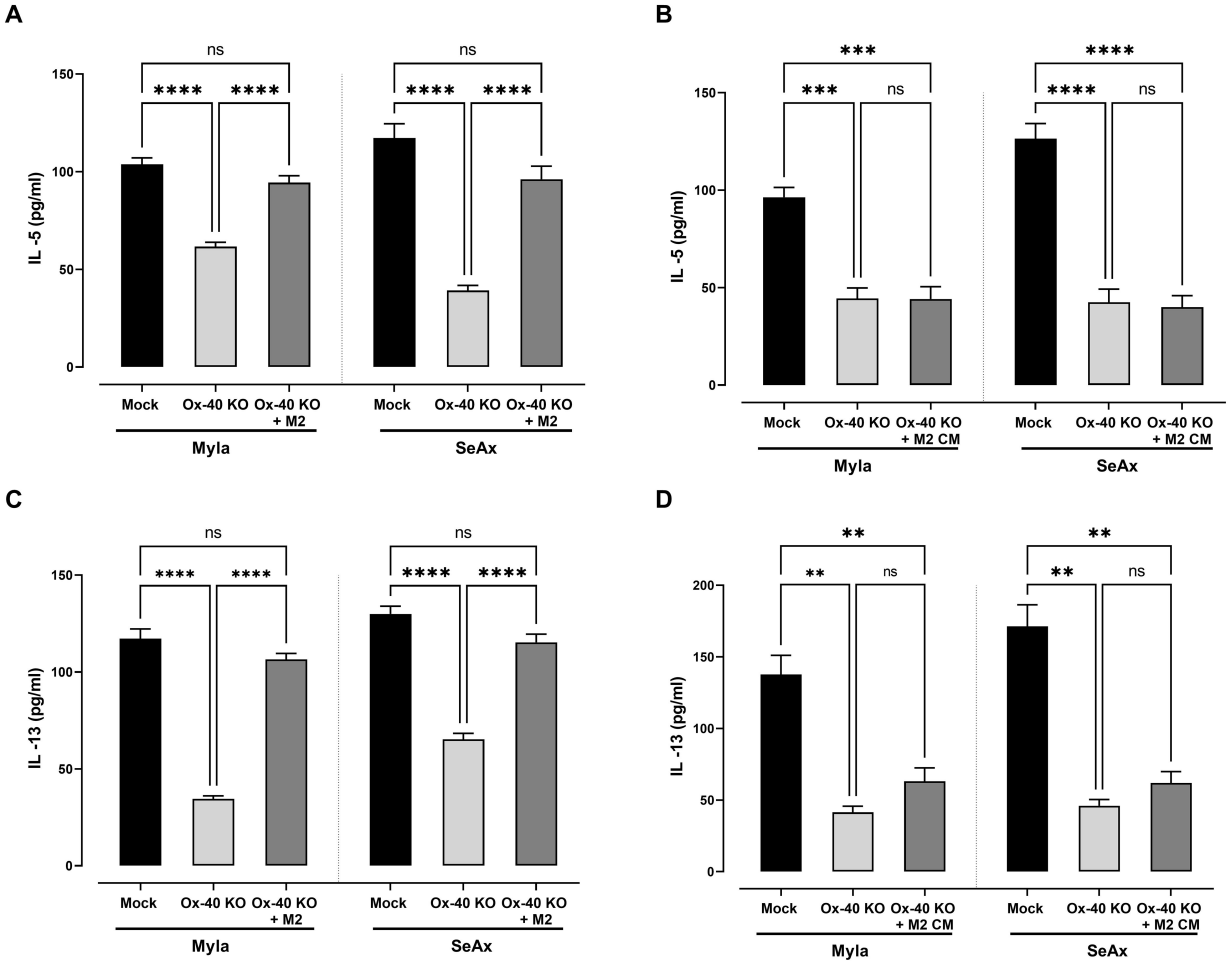
M2-Macrophages Restore Th2 Cytokine Expression in OX-40 KO CTCL cells. Decrease in **(A)** IL-5 and **(C)** IL-13 in OX-40 KO CTCL cells was restored in the presence of M2 macrophages but not in CM experiments **(B, D)**. Data are presented as mean ± SEM of three independent experiments. MyLa: 9 embryos, n=3 independent experiments; SeAx: 9 embryos, n=3 independent experiments; ****p<0.0001; ** p<0.01; ns: not significant (Brown-Forsythe and Welch ANOVA tests with Dunnett’s T3 multiple comparison test). KO, knockout; M, macrophage.

In OX-40 knockout CTCL cells, the production of both IL-5 (**Figure 6A**) and IL-13 (**Figure 6C**) was significantly reduced compared to that in controls (p <0.0001), highlighting OX-40’s role in regulating the pro-tumor cytokine network in CTCL. Interestingly, in the presence of M2 macrophages, the production of IL-13 and IL-5 was restored. To clarify whether the effect of M2 macrophages on cytokine secretion is mediated by soluble factors or requires direct cell–cell contact, we conducted conditioned medium (CM) experiments (**Figures 6B, D**). IL-5 and IL-13 in OX-40 KO CTCL cells were restored in the presence of M2 macrophage, but CM-derived from macrophages was not sufficient to restore cytokine secretion. Collectively, these findings suggest that the crosstalk between OX-40–deficient tumor cells and M2 macrophages is primarily dependent on cell–cell contact rather than being driven exclusively by paracrine signaling.

### 3.5 OX-40 affects lymphangiogenesis but not angiogenesis

**Figure 7:**
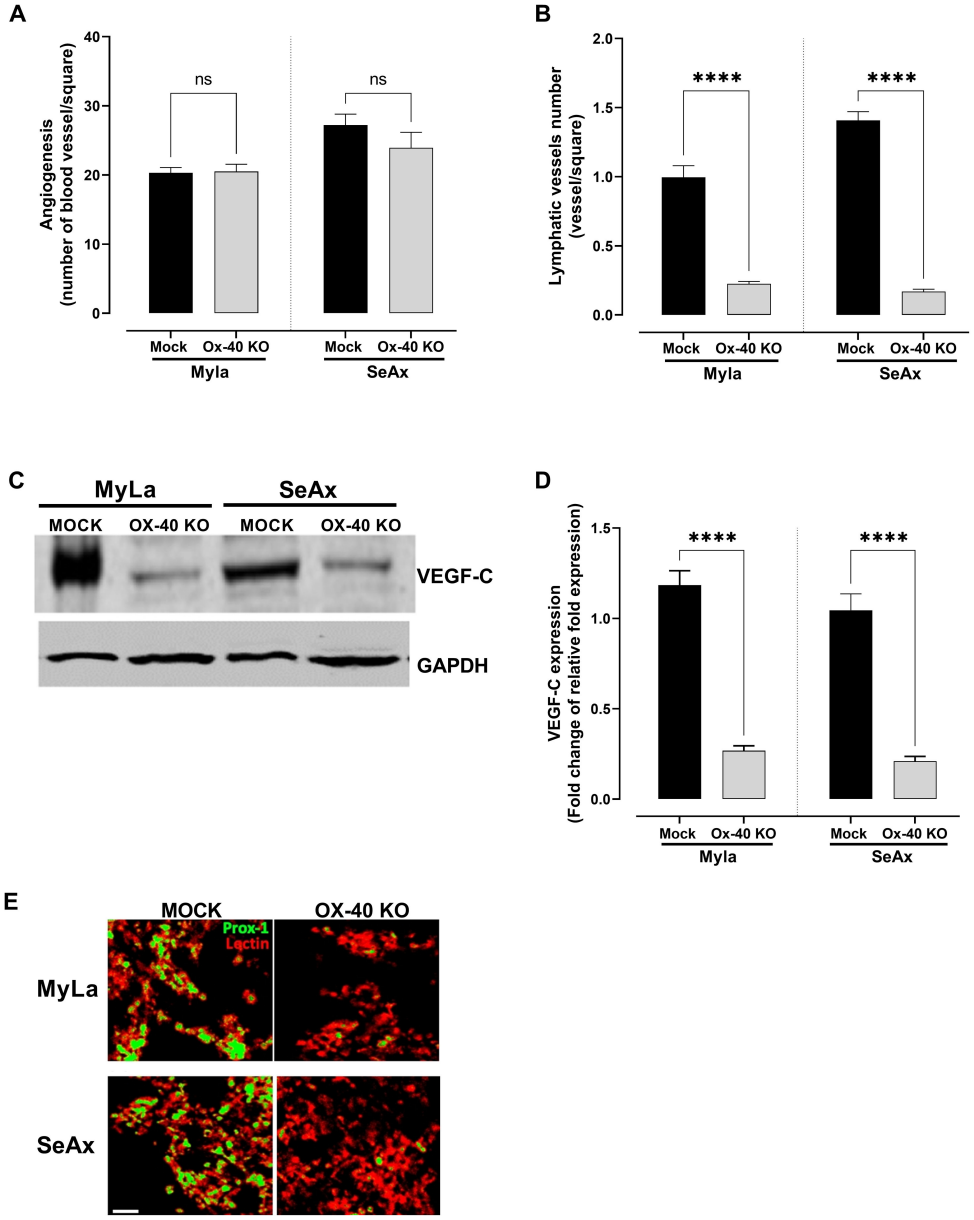
The effect of OX-40 on CTCL cells in angiogenesis and lymphangiogenesis. Measurement of **(A)** blood (unpaired t-test with Welch’s correction); and **(B)** lymphatic vessel formation (Mann-Whitney U test). VEGF-C expression by **(C)** Western blotting (unpaired t-test with Welch’s correction), and **(D)** RT-PCR (unpaired t-test with Welch’s correction). **(E)** Immunofluorescence staining of angiogenesis and lymphangiogenesis in CTCL tumors using the chick embryo CAM model (unpaired t-test with Welch’s correction). Images show blood vessels stained with lectin-Texas red and lymphatic vessels stained with anti-Prox1-FITC. Scale bars = 50 and 100 μm. Data are presented as mean ± SEM of two independent experiments. Angiogenesis MyLa: 10 embryos n=2 independent experiments; Angiogenesis SeAx: 9 embryos, n=2 independent experiments; Lymphatic vessel formation MyLa: 11 embryos, n=2 independent experiments; Lymphatic vessel formation SeAx: 9 embryos, n=2 independent experiments; RT-qPCR – 10 embryos in n=2 independent experiments; ns: not significant, ****p<0.0001; ns: not significant. KO, knockout; CAM, chorioallantoic membrane; VEGF, vascular endothelial growth factor.

The role of OX-40 in angiogenesis was further investigated, revealing that OX-40 did not significantly influence CTCL cell-induced angiogenesis. Evaluation using the CAM model showed no substantial difference in blood vessel formation between OX-40 knockout CTCL cells and their unmodified counterparts, as depicted in **Figure 7A**. Although a slight reduction in blood vessel formation was observed, the difference was not statistically significant, suggesting that OX-40 may not be involved in the regulation of angiogenesis. Therefore, we investigated the role of OX-40 in lymphangiogenesis. OX-40 knockout notably blocked lymphangiogenesis, showing less lymphatic vessel formation (**Figure 7B**) and decreased expression of VEGF-C protein (**Figure 7C**, **Supplementary Figure S6**) and mRNA levels (**Figure 7D**). Immunofluorescence analysis of Prox-1, a marker of lymphatic endothelial cells, and lectin, a marker of blood vessels and capillaries, demonstrated a significant reduction in lymphatic vessel formation in OX-40 knock out CTCL cells (**Figure 7E**). Moreover, decreased VEGF-C expression in OX-40 knockout CTCL cells further validated the role of OX-40 in lymphangiogenesis. This suggests that OX-40 promotes lymphangiogenesis, which may facilitate lymphatic metastasis in CTCL.

## 4 Discussion

Our study provides functional *in vivo* evidence that OX-40 drives spontaneous intravasation and distal metastasis of CTCL cells via ERK using a chick embryo CAM spontaneous metastasis model. In particular, we used a CAM model to monitor tumor behavior and a CRISPR-based knockout model in CTCL cells to provide important insights into the role of OX-40 in CTCL progression. Our results demonstrate a dramatic reduction in tumor weight, transendothelial migration, and distal metastasis upon inducing CRISPR-modification of OX-40 expression in CTCL cells through the ERK1/2 pathway. We also highlight the use of CRISPR/Cas9 loss-of-function and rescue experiments, the serial selection of highly-disseminating sublines, the demonstration that M2 macrophages restore Th2 cytokines and transendothelial migration in OX-40 KO cells, and the selective promotion of lymphangiogenesis (VEGF-C) rather than angiogenesis. These results not only provide novel insights into CTCL dissemination but also serve as the basis for future therapeutic strategies focusing on OX-40 modulation in a variety of malignancies.

The CAM assay (15), a rapid and ethical *in vivo* model, enabled the visualization of transendothelial migration, metastatic dissemination, and tumor reduction in OX-40–modulated CTCL cells. We observed that highly disseminated CTCL cells overexpressed OX-40 and showed increased phosphorylation of ERK. In our OX-40 knockout CTCL cell lines, the reduction in ERK signaling suggests that OX-40 is a critical upstream regulator of the ERK pathway, which drives tumor growth and dissemination in CTCL. We have found that OX-40 interaction causes the ERK signaling cascade to be activated in CTCL cells through the increase in phosphorylation of ERK and its effectors RSK and ELK. This is important since both RSK and ELK transcription factors do not only control gene programs that regulate proliferation, survival, migration, and invasion, but also these are key processes that define the tumor microenvironment (23, 24). It has been demonstrated previously that the RSK family can mediate tumor invasion and metastasis through the regulation of cytoskeletal dynamics, EMT and survival signaling, and aberrant ERK/ELK1 can mediate lymphoma progression and spreading. Although dysregulation of ERK/MAPK pathway is already established in CTCL, the downstream effectors like RSK are sparsely reported. Nonetheless, anti-ELK1 expression has been found to be high in Mycosis Fungoides samples (25) as well as in spatial transcriptome profiling of melanoma (26), which proves the possible relevance of ELK1 in the pathogenesis of CTCL. Though direct association of the role of ERK1/2-ELK1 signaling with metastasis in lymphoma has not been made, given its capacity to promote transcriptional programs that promote tumor growth and tumor aggressiveness, one might consider analogous processes are operating in the case of CTCL. Altogether, these findings validate a hypothesis of OX-40 signaling promoting ERK-mediated RSK and ELK activation to strengthen tumor-microenvironment interactions and increase metastatic potential in CTCL.

Our results highlight the importance of OX-40 in promoting CTCL progression and support its role as a potential therapeutic target. The findings of the chick embryo model are consistent with the alternate use of OX-40 signaling in other cancers, where it also modulates tumor behavior.

The ERK1/2 pathway is a key signaling cascade that regulates cellular proliferation and survival. It has also been implicated in several malignancies including CTCL. Its activation has been linked to tumor cell migration and invasion, implying that its regulation by OX-40 could be a novel mechanism important for CTCL progression (27, 28). Similarly, it have been shown that OX-40 agonists enhance T cell infiltration and inhibit tumor growth in melanoma (29, 30). Moreover, the significance of the ERK1/2 pathway has been demonstrated in breast cancer, as its activation correlates with metastatic potential (31). These data imply that crosstalk between OX-40 and the ERK1/2 pathway could be a conserved mechanism in various malignancies, and targeting OX-40 represents a promising therapeutic avenue.

OX-40 is a co-stimulatory molecule that modulates immune responses, maintains balance in the TME. It has become an attractive therapeutic target because it can enhance T cell activation, antagonize Treg suppression, and augment effector functions (8). Preclinical studies have shown that OX-40 agonists exert potent antitumor activity in several cancer types, including melanoma and breast cancer (32, 33), highlighting the therapeutic potential of OX-40 for CTCL. Moreover, recent clinical trials of OX40 agonists in cancer have demonstrated their potential to enhance T-cell activation and anti-tumor immunity. Agents such as BMS-986178, MEDI0562, and ivuxolimab (PF-04518600) have been tested in Phase I/II studies across various advanced solid tumors, including lung, renal, and bladder cancers. These studies consistently show that OX40 agonists are generally well tolerated and induce pharmacodynamic evidence of T-cell activation. Combination approaches, for example BMS-986178 with TLR9 agonist SD-101 and low-dose radiation, aim to further amplify anti-tumor responses. While these early-phase trials are encouraging, ongoing research is needed to determine their clinical efficacy and optimal use in cancer therapy (34–40). OX-40 signaling modulation could promote effector T cell entry into the tumor environment, proliferation, and pro-inflammatory cytokine production, which are critical components of effective tumor rejection (28, 30, 41). OX-40 signaling can augment combination therapies (CTLA-4 blockade plus 4-1BB activation), with increased T-cell infiltration and cytokine production reported (30). These data imply that combinational approaches of this type should be explored to improve the therapeutic efficacy of CTCL. High OX-40 expression in CTCL has been correlated with T cell activation and growth, which are required for anti-tumor immunity (12, 32, 33).

Additionally, we showed that M2 macrophage function was modulated by OX-40 activity in transendothelial migration, CAM intravasation, and distal metastatic events of tumor progression, which remain important factors in CTCL pathology. Wu et al. demonstrated that the depletion of M2-like tumor-associated macrophages delayed CTCL development *in vivo* (42). The presence of M2 macrophages in the affected skin of MF patients has been clinically correlated with patient prognosis, indicating that tumor-associated macrophages (TAMS) may play a major role in the pathophysiology of MF (42). Our findings underline the significant role of OX-40 signaling in the TME, possibly through M2 macrophages, which secrete factors that promote tumor cell migration and invasion by modulating the cytokine milieu and enhancing the tumor-supportive properties of the TME. OX-40 has a critical impact on tumor behavior, whereas M2 macrophages promote tumor growth and metastasis via numerous mechanisms. Our co-culture experiments demonstrated that M2 macrophages were able to restore cytokine secretion and enhance the metastatic potential of OX-40–deficient tumor cells. Interestingly, conditioned medium from M2 macrophages failed to reproduce these effects, indicating that soluble factors alone are insufficient to drive this phenotype. Instead, our data strongly suggest that direct cell–cell interactions are required for the pro-tumoral activity of M2 macrophages in this setting. This observation is consistent with previous reports showing that macrophage–tumor cell interactions can promote invasion and metastasis through mechanisms involving membrane-bound ligands, adhesion molecules, and the formation of specialized contact structures such as tunneling nanotubes (43). In line with these findings, our study emphasizes the importance of physical crosstalk between tumor cells and the immunosuppressive microenvironment in driving metastatic progression. Targeting these contact-dependent interactions, in addition to paracrine signaling pathways, may therefore represent an effective therapeutic strategy to disrupt macrophage-mediated tumor promotion. When we analyzed the changes in the phosphorylation of signaling pathways, we found that phosphorylated ERK1/2 was reduced as well as downstream targets. Recent studies have shown that OX-40 signaling mediates crosstalk between tumor cells and the TME, including macrophages. M2 macrophages are generally associated with activities that favor tumors and have been shown to favor transendothelial migration of tumor cells. For example, Roh-Johnson et al. showed that tumor cell contact with macrophages activates RhoA GTPase signaling, which is necessary for tumor cell intravasation (44). This implies that M2-like macrophages can promote the transit of CTCL cells through an endothelial barrier, a critical step in the process of metastasis. Pignatelli et al. showed the essential roles of Notch1 and MenaINV in the formation of invadopodia, which promote transendothelial migration (45). Such interactions between OX-40 and M2-like macrophages may further upregulate these processes, promoting the metastatic potential of CTCL. For example, paracrine signaling from macrophages enhances tumor cell intravasation and metastasis in other malignancies, such as breast cancer (46).

Importantly, the crosstalk between OX-40 expressing CTCL cells and M2 macrophages can also impact cytokine profiles; specifically, IL-5 and IL-13 expression in OX-40 knockout CTCL cells was restored by M2 macrophages. This finding was particularly interesting because IL-5 and IL-13 are both linked to Th2 immune responses, which can enhance tumor growth and survival (47). M2 macrophages promote a cytokine microenvironment that benefits CTCL cell survival and proliferation. In other malignancies, M2 macrophages have been implicated in the re-establishment or enhancement of cytokine expression, which supports tumorigenesis. M2 macrophages can secrete IL-10 and TGF-β mediators, which promote tumor progression and immune evasion (48, 49). This suggests a potential role for M2 macrophages in modulating the immune landscape in CTCL via cytokine signaling.

The regulation of the cytokine profile, specifically of Th2-associated cytokines such as IL-13 and IL-5, has also been linked to OX-40. IL-13, which is known to promoting the Th-2 phenotype in both malignant CTCL cells and non-malignant T cells, along with IL-5, plays a crucial role in tumor progression. This reduction in Th2 cytokines suggests that targeting OX-40 may disrupt the tumor-supportive immune microenvironment, thereby impairing tumor growth and dissemination. This finding further strengthens the rationale for using OX-40 inhibition as a therapeutic strategy to interfere with cytokine-mediated CTCL progression.

Interestingly, our findings revealed that OX-40-expressing CTCL cells modulate lymphangiogenesis rather than angiogenesis. The process of formation of new lymphatic vessels, known as lymphangiogenesis, is critical for the dissemination of malignant cells, especially in lymphatic metastasis. Given that lymphatic spread is a key feature of advanced CTCL, we showed that inhibition of OX-40 could potentially limit tumor dissemination through the lymphatic system, providing a novel therapeutic avenue. Previous studies have proposed that tumor cells secrete factors that stimulate lymphatic endothelial cell proliferation and migration, thereby promoting lymphangiogenesis (50). TAMs have recently been shown to promote tumor lymphangiogenesis followed by elevated VEGF-C expression (51). Angiogenesis is typically regulated by alternative signaling networks such as VEGF. We showed that OX-40-expressing CTCL cells selectively promote lymphangiogenesis, most likely by modifying lymphatic endothelial signaling pathways such as VEGF-C. The development of lymphangiogenesis versus angiogenesis by OX-40 CTCL cells indicates a novel mechanism whereby OX-40 CTCL cells preferentially spread through lymphatics in comparison to blood. This has also been  observed in other cancers, whereby distinct tumor cell characteristics influence the preference for lymphatic or blood vessel formation (52). The association between OX-40/M2 macrophages and the metastatic nature of CTCL cells provides further understanding of the role of the TME and its influence on the metastatic spread of CTCL cells. Furthermore, the modulation of transendothelial migration, cytokine expression, and lymphangiogenesis suggests potential therapeutic targets in CTCL and possibly in other malignancies.

One major limitation of the present work is the fact that the chick embryo CAM system does not fully recapitulate the complexity of an intact mammalian immune system. Although the CAM is used to study dissemination of tumor cells, intravasation and colonization *in vivo*, it is not able to embrace the entire spectrum of adaptive immune interactions that determine the progression of CTCL in patients. Therefore, we conclude on the cell-intrinsic and microenvironmental effects of OX-40 which are maintained in this model, but further studies in the immunocompetent murine systems will be necessary to completely understand the role played by OX-40 in tumor-immune crosstalk in CTCL. Further research is required to clarify the exact mechanisms involved in the role of OX-40 in the progression of CTCL, as well as to determine its potential synergistic effects with other available therapies. These results indicate that modulation of the OX-40 signaling pathway may represent a new therapeutic target for the treatment of CTCL.

In conclusion, our study illustrates that OX-40 is a significant mediator of dissemination and microenvironmental interactions of CTCL cells in a chick embryo in a preclinical environment. A possible key regulator of CTCL development is the OX-40 signaling axis. Through ERK activation, OX-40 promotes tumor development, metastasis, and Th2 cytokine production while affecting the immunosuppressive characteristics of the TME, such as lymphangiogenesis and macrophage activity. These findings confirm the necessity of OX-40-targeted intervention for further assessment in more rigorous immunocompatibility models and eventually in clinical trials, although conclusive clinical treatment phenotypes are yet to be determined.

## Data Availability

The raw data supporting the conclusions of this article will be made available by the authors, without undue reservation.

## References

[1] Querfeld C Guitart J Kuzel TM Rosen ST. Primary cutaneous lymphomas: A review with current treatment options. Blood Rev. (2003) 17:131–42. doi: 10.1016/S0268-960X(03)00004-3, PMID: 12818223

[2] Willemze R Jaffe ES Burg G Cerroni L Berti E Swerdlow SH. WHO-EORTC classification for cutaneous lymphomas. Blood. (2005) 105:3768–85. doi: 10.1182/blood-2004-09-3502, PMID: 15692063

[3] Willemze R Cerroni L Kempf W Berti E Facchetti F Swerdlow SH. The 2018 update of the WHO-EORTC classification for primary cutaneous lymphomas. Blood. (2019) 133:1703–14. doi: 10.1182/blood-2018-11-881268, PMID: 30635287 PMC6473500

[4] Arulogun SO Prince HM Ng J Lade S Ryan GF Blewitt O. Long-term outcomes of patients with advanced-stage cutaneous T-cell lymphoma and large cell transformation. Blood. (2008) 112:3082–7. doi: 10.1182/blood-2008-05-154609, PMID: 18647960

[5] Khan S Sawas A. Antibody-directed therapies: toward a durable and tolerable treatment platform for CTCL. Front Oncol. (2019) 9. doi: 10.3389/fonc.2019.00645, PMID: 31417860 PMC6683760

[6] Pavlidis A Piperi C Papadavid E. Novel therapeutic approaches for cutaneous T cell lymphomas. Expert Rev Clin Immunol. (2021) 17:629–41. doi: 10.1080/1744666X.2021.1919085, PMID: 33890833

[7] Oka T Miyagaki T. Novel and future therapeutic drugs for advanced mycosis fungoides and sézary syndrome. Front Med. (2019) 6. doi: 10.3389/fmed.2019.00116, PMID: 31192214 PMC6548851

[8] Guglielmo A Borghi A Zengarini C Piraccini BM Corazza M Pileri A. OX40–OX40L axis in cutaneous T-cell lymphomas: pathogenic, prognostic, and potential therapeutic perspectives. Biomolecules. (2025) 15:715. doi: 10.3390/biom15050715, PMID: 40427608 PMC12109069

[9] Kim BS Kim JY Kim EJ Lee JG Joo DJ Huh KH. Role of thalidomide on the expression of OX40, 4-1BB, and GITR in T cell subsets. Transplant Proc. (2016) 48:1270–4. doi: 10.1016/j.transproceed.2015.12.088, PMID: 27320601

[10] Wyzgol A Müller N Fick A Munkel S Grigoleit GU Pfizenmaier K. Trimer stabilization, oligomerization, and antibody-mediated cell surface immobilization improve the activity of soluble trimers of CD27L, CD40L, 41BBL, and glucocorticoid-induced TNF receptor ligand. J Immunol. (2009) 183:1851–61. doi: 10.4049/jimmunol.0802597, PMID: 19596991

[11] Vieyra-Garcia P Crouch JD O’Malley JT Seger EW Yang CH Teague JE. Benign T cells drive clinical skin inflammation in cutaneous T cell lymphoma. JCI Insight. (2019) 4(1):e124233. doi: 10.1172/jci.insight.124233, PMID: 30626755 PMC6485670

[12] Kawana Y Suga H Kamijo H Miyagaki T Sugaya M Sato S. Roles of OX40 and OX40 ligand in mycosis fungoides and sézary syndrome. Int J Mol Sci. (2021) 22(22):12576–90. doi: 10.3390/ijms222212576, PMID: 34830466 PMC8617822

[13] Querfeld C Leung S Myskowski PL Curran SA Goldman DA Heller G. Primary T cells from cutaneous T-cell lymphoma skin explants display an exhausted immune checkpoint profile. Cancer Immunol Res. (2018) 6:900–9. doi: 10.1158/2326-6066.CIR-17-0270, PMID: 29895574 PMC6074045

[14] Karagianni F Piperi C Casar B de la Fuente-Vivas D García-Gómez R Lampadaki K. Combination of resminostat with ruxolitinib exerts antitumor effects in the chick embryo chorioallantoic membrane model for cutaneous T cell lymphoma. Cancers (Basel). (2022) 14(4):1070–91. doi: 10.3390/cancers14041070, PMID: 35205818 PMC8870185

[15] Merlos Rodrigo MA Casar B Michalkova H Jimenez Jimenez AM Heger Z Adam V. Extending the applicability of in ovo and ex ovo chicken chorioallantoic membrane assays to study cytostatic activity in neuroblastoma cells. Front Oncol. (2021) 11. doi: 10.3389/fonc.2021.707366, PMID: 34540673 PMC8440826

[16] Crespo P Casar B. The chick embryo chorioallantoic membrane as an *in vivo* model to study metastasis. BIO-PROTOCOL. (2016) 6:1962–73. doi: 10.21769/BioProtoc.1962

[17] Cong L Ran FA Cox D Lin S Barretto R Habib N. Multiplex genome engineering using CRISPR/Cas systems. Sci (80-). (2013) 339:819–23. doi: 10.1126/science.1231143, PMID: 23287718 PMC3795411

[18] Ran FA Hsu PD Wright J Agarwala V Scott DA Zhang F. Genome engineering using the CRISPR-Cas9 system. Nat Protoc. (2013) 8:2281–308. doi: 10.1038/nprot.2013.143, PMID: 24157548 PMC3969860

[19] Greiner D Scott TM Olson GS Aderem A Roh-Johnson M Johnson JS. Genetic modification of primary human myeloid cells to study cell migration, activation, and organelle dynamics. Curr Protoc. (2022) 2(8):e514 . doi: 10.1002/cpz1.514, PMID: 36018279 PMC9476234

[20] Deryugina EI Quigley JP. Chapter 2. Chick embryo chorioallantoic membrane models to quantify angiogenesis induced by inflammatory and tumor cells or purified effector molecules. Methods Enzymol. (2008) 444:21–41. doi: 10.1016/S0076-6879(08)02802-4, PMID: 19007659 PMC2699944

[21] Schindelin J Arganda-Carreras I Frise E Kaynig V Longair M Pietzsch T. Fiji: An open-source platform for biological-image analysis. Nat Methods. (2012) 9:676–82. Available online at: https://www.nature.com/articles/nmeth.2019 (Accessed May 28, 2025)., PMID: 22743772 10.1038/nmeth.2019 PMC3855844

[22] Bayne LJ Beatty GL Jhala N Clark CE Rhim AD Stanger BZ. Tumor-derived granulocyte-macrophage colony-stimulating factor regulates myeloid inflammation and T cell immunity in pancreatic cancer. Cancer Cell. (2012) 21:822–35. doi: 10.1016/j.ccr.2012.04.025, PMID: 22698406 PMC3575028

[23] Yang WS Caliva MJ Khadka VS Tiirikainen M Matter ML Deng Y. RSK1 and RSK2 serine/threonine kinases regulate different transcription programs in cancer. Front Cell Dev Biol. (2023) 10:1015665. doi: 10.3389/fcell.2022.1015665, PMID: 36684450 PMC9845784

[24] Kalampounias G Androutsopoulou T Katsoris P. Mechanistic insights and clinical implications of ELK1 in solid tumors: A narrative review. Cells. (2025) 14:1257. Available online at: https://www.mdpi.com/2073-4409/14/16/1257/htm (Accessed September 23, 2025)., PMID: 40862737 10.3390/cells14161257 PMC12384596

[25] Seçme M Dodurga Y Demirkan NÇ Kaçar N Günel NS Açıkbaş İ. Determination of T-cell clonality and expression profiles of Toll-like receptors signaling pathway genes and related miRNAs in patients with mycosis fungoides. Gene. (2024) 891:147825. Available online at: https://www.sciencedirect.com/science/article/abs/pii/S0378111923006662 (Accessed September 19, 2025)., PMID: 37748629 10.1016/j.gene.2023.147825

[26] Kiuru M Kriner MA Wong S Zhu G Terrell JR Li Q. High-Plex Spatial RNA Profiling Reveals Cell Type–Specific Biomarker Expression during Melanoma Development. J Invest Dermatol. (2022) 142:1401–1412.e20. Available online at: https://www.jidonline.org/action/showFullText?pii=S0022202X21023708 (Accessed January 20, 2025)., PMID: 34699906 10.1016/j.jid.2021.06.041 PMC9714472

[27] Massarelli E Lam VK Parra ER Rodriguez-Canales J Behrens C Diao L. High OX-40 expression in the tumor immune infiltrate is a favorable prognostic factor of overall survival in non-small cell lung cancer. J Immunother Cancer. (2019) 7(1):351–9. doi: 10.1186/s40425-019-0827-2, PMID: 31843013 PMC6915970

[28] Dushyanthen S Teo ZL Caramia F Savas P Mintoff CP Virassamy B. Agonist immunotherapy restores T cell function following MEK inhibition improving efficacy in breast cancer. Nat Commun. (2017) 8:1–18. Available online at: https://www.nature.com/articles/s41467-017-00728-9 (Accessed January 20, 2025)., PMID: 28928458 10.1038/s41467-017-00728-9 PMC5605577

[29] Burocchi A Pittoni P Gorzanelli A Colombo MP Piconese S. Intratumor OX40 stimulation inhibits IRF1 expression and IL-10 production by Treg cells while enhancing CD40L expression by effector memory T cells. Eur J Immunol. (2011) 41:3615–26. doi: 10.1002/eji.201141700, PMID: 22229156

[30] Curran MA Kim M Montalvo W Al-Shamkhani A Allison JP. Combination CTLA-4 blockade and 4-1BB activation enhances tumor rejection by increasing T-cell infiltration, proliferation, and cytokine production. PloS One. (2011) 6(4):e19499. doi: 10.1371/journal.pone.0019499, PMID: 21559358 PMC3085474

[31] Sun J Yi S Qiu L Fu W Wang A Liu F. SATB1 defines a subtype of cutaneous CD30+ Lymphoproliferative disorders associated with a T-helper 17 cytokine profile. J Invest Dermatol. (2018) 138:1795–804. doi: 10.1016/j.jid.2018.02.028, PMID: 29510190

[32] Curti BD Kovacsovics-Bankowski M Morris N Walker E Chisholm L Floyd K. OX40 is a potent immune-stimulating target in late-stage cancer patients. Cancer Res. (2013) 73:7189–98. doi: 10.1158/0008-5472.CAN-12-4174, PMID: 24177180 PMC3922072

[33] Weinberg AD Morris NP Kovacsovics-Bankowski M Urba WJ Curti BD. Science gone translational: the OX40 agonist story. Immunol Rev. (2011) 244:218–31. doi: 10.1111/j.1600-065X.2011.01069.x, PMID: 22017441 PMC3622727

[34] Davis EJ Martin-Liberal J Kristeleit R Cho DC Blagden SP Berthold D. First-in-human phase I/II, open-label study of the anti-OX40 agonist INCAGN01949 in patients with advanced solid tumors. J Immunother Cancer. (2022) 10:4235. Available online at: https://jitc.bmj.com/content/10/10/e004235 (Accessed September 19, 2025)., PMID: 36316061 10.1136/jitc-2021-004235 PMC9628691

[35] Shree T Czerwinski D Haebe S Sathe A Grimes S Martin B. A phase I clinical trial adding OX40 agonism to *in situ* therapeutic cancer vaccination in patients with low-grade B-cell lymphoma highlights challenges in translation from mouse to human studies. Clin Cancer Res. (2025) 31:868–80. doi: 10.1158/1078-0432.CCR-24-2770, PMID: 39745391 PMC11922159

[36] Hong WX Sagiv-Barfi I Czerwinski DK Sallets A Levy R. Neoadjuvant intratumoral immunotherapy with TLR9 activation and anti-OX40 antibody eradicates metastatic cancer. Cancer Res. (2022) 82:1396–408. doi: 10.1158/0008-5472.CAN-21-1382, PMID: 35135810 PMC8983569

[37] Gutierrez M Moreno V Heinhuis KM Olszanski AJ Spreafico A Ong M. OX40 agonist BMS-986178 alone or in combination with nivolumab and/or ipilimumab in patients with advanced solid tumors. Clin Cancer Res. (2021) 27:460–72. doi: 10.1158/1078-0432.CCR-20-1830, PMID: 33148673

[38] Postel-Vinay S Lam VK Ros W Bauer TM Hansen AR Cho DC. First-in-human phase I study of the OX40 agonist GSK3174998 with or without pembrolizumab in patients with selected advanced solid tumors (ENGAGE-1). J Immunother Cancer. (2023) 11:e005301. doi: 10.1136/jitc-2022-005301, PMID: 36927527 PMC10030671

[39] Glisson BS Leidner RS Ferris RL Powderly J Rizvi NA Keam B. Safety and clinical activity of MEDI0562, a humanized OX40 agonist monoclonal antibody, in adult patients with advanced solid tumors. Clin Cancer Res. (2020) 26:5358–67. Available online at: https://aacrjournals.org/clincancerres/article/26/20/5358/82745/Safety-and-Clinical-Activity-of-MEDI0562-a (Accessed September 19, 2025)., PMID: 32816951 10.1158/1078-0432.CCR-19-3070

[40] Zhou H Ma Y Li Y Tang L Guo Y Yuan G. Anti-OX40 antibody BAT6026 in patients with advanced solid tumors: A multi-center phase I study. iScience. (2025) 28:112270. Available online at: https://www.sciencedirect.com/science/article/pii/S2589004225005310 (Accessed September 19, 2025)., PMID: 40520088 10.1016/j.isci.2025.112270 PMC12164209

[41] Thapa B Kato S Nishizaki D Miyashita H Lee S Nesline MK. OX40/OX40 ligand and its role in precision immune oncology. Cancer Metastasis Rev. (2024) 43:1001–13. doi: 10.1007/s10555-024-10184-9, PMID: 38526805 PMC11300540

[42] Wu X Schulte BC Zhou Y Haribhai D Mackinnon AC Plaza JA. Depletion of M2-like tumor-associated macrophages delays cutaneous T-cell lymphoma development *in vivo* . J Invest Dermatol. (2014) 134:2814–22. doi: 10.1038/jid.2014.206, PMID: 24780929

[43] Hanna SJ McCoy-Simandle K Leung E Genna A Condeelis J Cox D. Tunneling nanotubes, a novel mode of tumor cell–macrophage communication in tumor cell invasion. J Cell Sci. (2019) 132:jcs223321. doi: 10.1242/jcs.223321, PMID: 30659112 PMC6382011

[44] Roh-Johnson M Bravo-Cordero JJ Patsialou A Sharma VP Guo P Liu H. Macrophage contact induces RhoA GTPase signaling to trigger tumor cell intravasation. Oncogene. (2014) 33:4203–12. doi: 10.1038/onc.2013.377, PMID: 24056963 PMC3962803

[45] Pignatelli J Bravo-Cordero JJ Roh-Johnson M Gandhi SJ Wang Y Chen X. Macrophage-dependent tumor cell transendothelial migration is mediated by Notch1/MenaINV-initiated invadopodium formation. Sci Rep. (2016) 6:6:37874–89. doi: 10.1038/srep37874, PMID: 27901093 PMC5129016

[46] Roussos ET Balsamo M Alford SK Wyckoff JB Gligorijevic B Wang Y. Mena invasive (MenaINV) promotes multicellular streaming motility and transendothelial migration in a mouse model of breast cancer. J Cell Sci. (2011) 124:2120. doi: 10.1242/jcs.086231, PMID: 21670198 PMC3113666

[47] Dhakal M Hardaway JC Guloglu FB Miller MM Hoeman CM Zaghouani AA. IL-13Rα1 is a surface marker for M2 macrophages influencing their differentiation and function. Eur J Immunol. (2014) 44:842–55. doi: 10.1002/eji.201343755, PMID: 24281978 PMC3959573

[48] Huang C Ou R Chen X Zhang Y Li J Liang Y. Tumor cell-derived SPON2 promotes M2-polarized tumor-associated macrophage infiltration and cancer progression by activating PYK2 in CRC. J Exp Clin Cancer Res. (2021) 40(1):304–21. doi: 10.1186/s13046-021-02108-0, PMID: 34583750 PMC8477524

[49] Fadhil M Abdul- Rasheed O Al-Naqqash M. Clinical value of peripheral blood M2/M1 like monocyte ratio in the diagnosis of breast cancer and the differentiation between benign and Malignant breast tumors. Acta Biochim Pol. (2019) 66:437–443. doi: 10.18388/abp.2019_2855, PMID: 31794652

[50] Cabrera RM Mao SPH Surve CR Condeelis JS Segall JE. A novel neuregulin - jagged1 paracrine loop in breast cancer transendothelial migration. Breast Cancer Res. (2018) 20(1):24–34. doi: 10.1186/s13058-018-0960-8, PMID: 29636067 PMC5894135

[51] Hwang I Kim JW Ylaya K Chung EJ Kitano H Perry C. Tumor-associated macrophage, angiogenesis and lymphangiogenesis markers predict prognosis of non-small cell lung cancer patients. J Transl Med. (2020) 18(1):443–58. doi: 10.1186/s12967-020-02618-z, PMID: 33228719 PMC7686699

[52] Harney AS Arwert EN Entenberg D Wang Y Guo P Qian BZ. Real-time imaging reveals local, transient vascular permeability, and tumor cell intravasation stimulated by TIE2hi macrophage-derived VEGFA. Cancer Discov. (2015) 5:932–43. doi: 10.1158/2159-8290.CD-15-0012, PMID: 26269515 PMC4560669

