# Supplementary material for: OX-40 signaling promotes tumorigenesis in CTCL by regulating ERK activation
Source: Front Immunol. 2025 Oct 27;16:1677140. doi: 10.3389/fimmu.2025.1677140 (PMC12597999; doi:10.3389/fimmu.2025.1677140)
Supplement: Supplementary file 1 [file DataSheet1.pdf]

Supplementary Material

OX-40 Signaling Promotes Tumorigenesis in CTCL by Regulating ERK Activation

Evangelia Papadavid*†, Fani Karagianni†, Eleni-Kyriaki Vetsika, Sara Valero-Díaz, Saire Edith Córdova-Hernández, Christos Daniil, Christina Piperi, Berta Casar*

† These authors contributed equally to this work and share first authorship

*** Correspondence:** Evangelia Papadavid: epapad@med.uoa.gr ; Berta Casar: casarb@unican.es

1 Supplementary Figures and Tables

1.1 Supplementary Figures

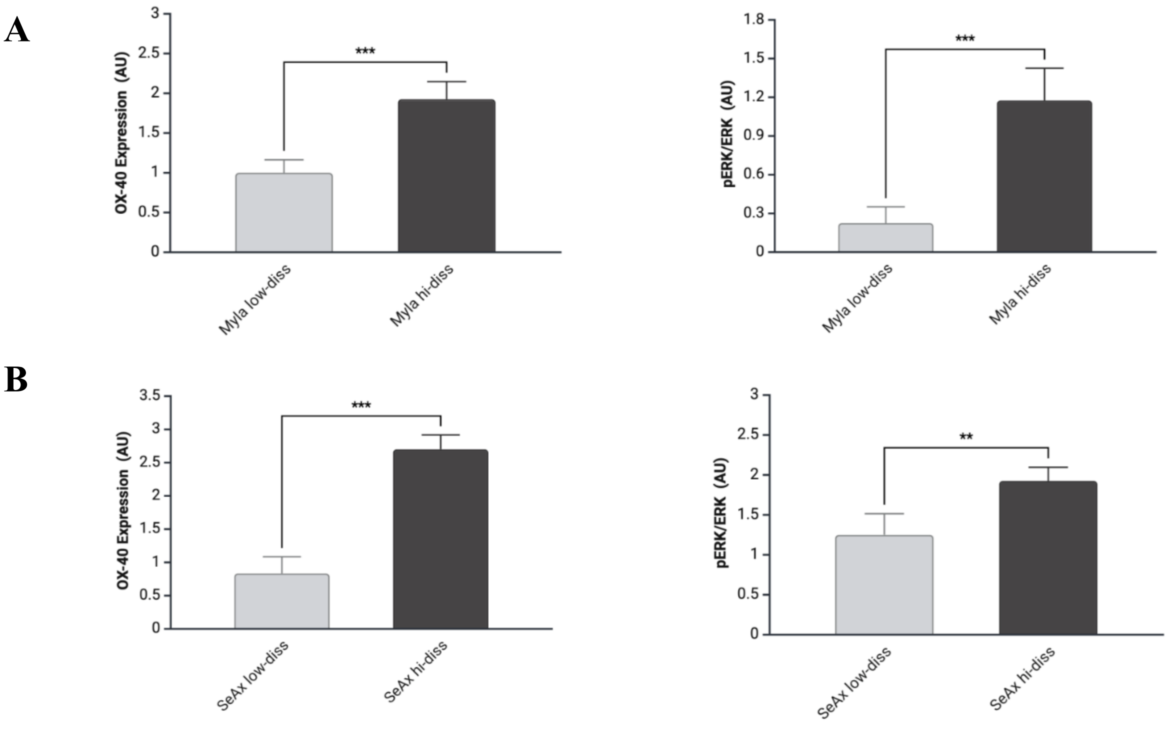

Supplementary Figure S1. Western Blot analysis quantification of high and low disseminated CTCL cells, **(A)** Myla and **(B)** SeAx for the OX- 40 expression and p-ERK activation (Tyr 204) (unpaired t-test with Welch's correction). Significance was defined as $p \leq 0.05$ and denoted as: ** $p < 0.01$, *** $p < 0.005$. (San Diego, CA, USA).

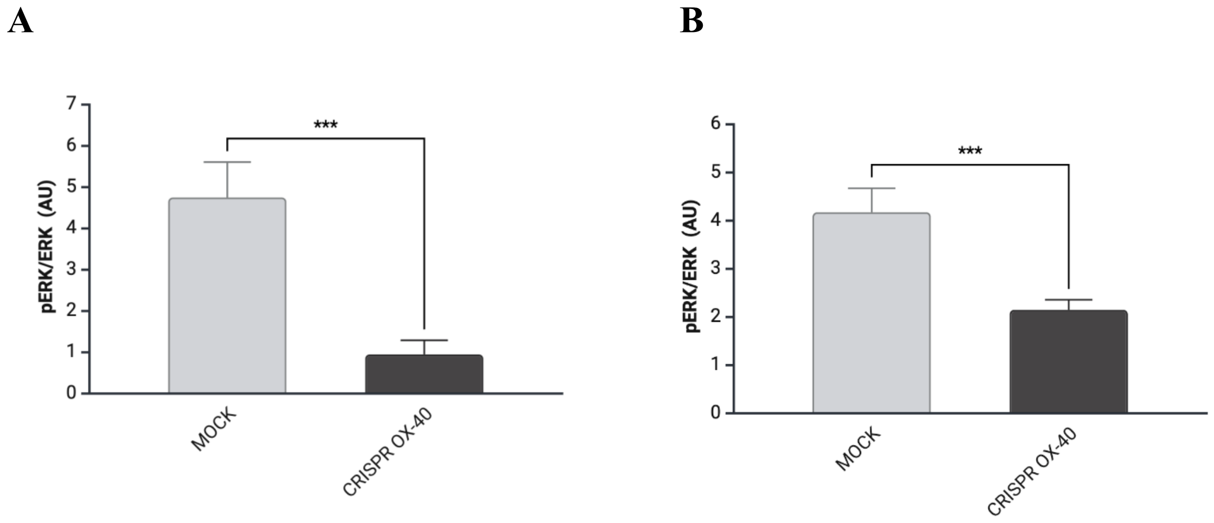

Supplementary Figure S2. Quantification of ERK1/2 phosphorylation in CAM grafted CTCL tumors **(A)** MyLa and (**B)** SeAx. Significance was defined as p ≤ 0.05 and denoted as: *** p < 0.005. Analysis was performed using GraphPad Prism 8 software (San Diego, CA, USA).

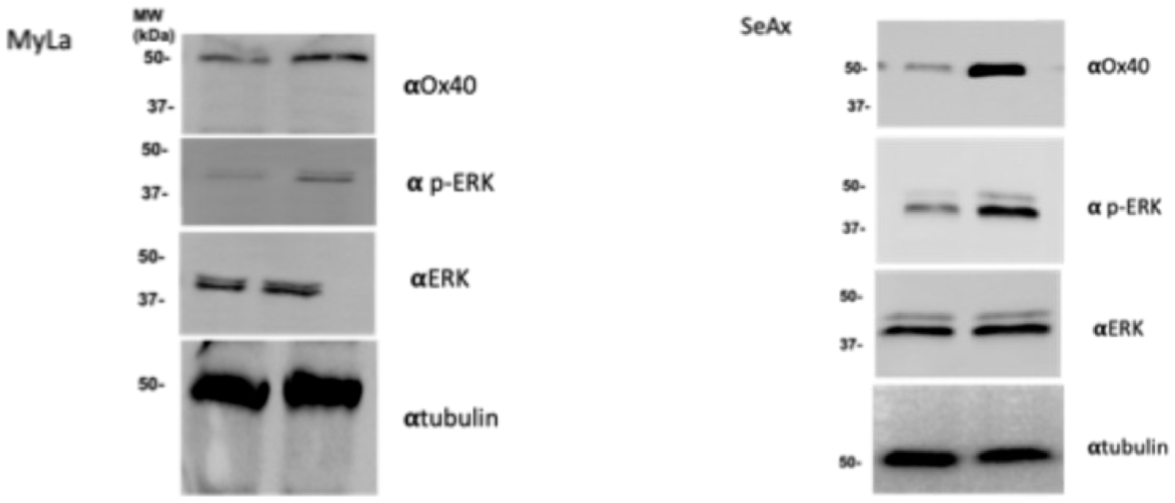

Supplementary Figure S3. Unprocessed images for Western Blot results corresponding to Figure 1

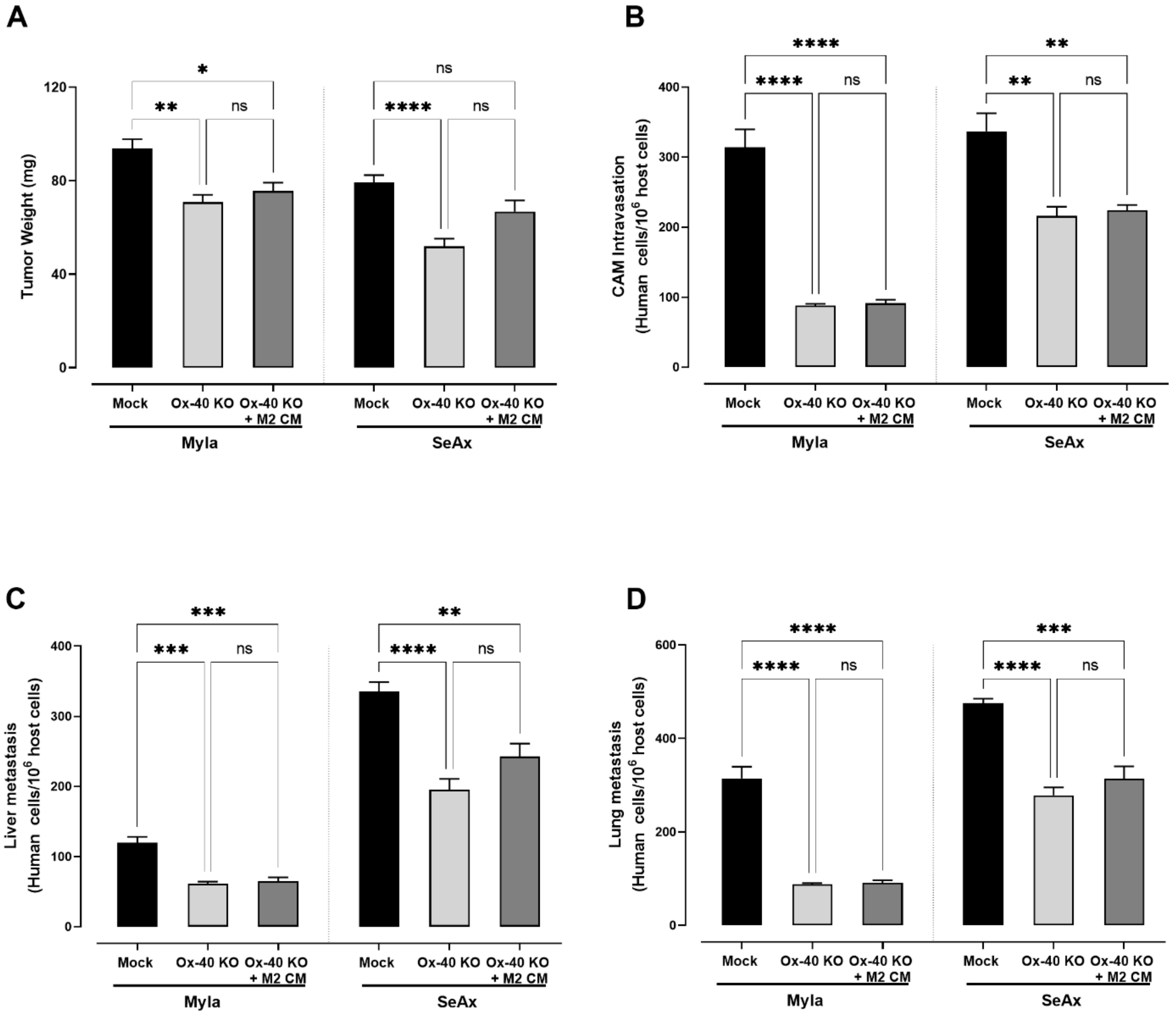

Supplementary Figure S4. The effect of OX-40 loss on CTCL cells under conditioned medium experiment in (A) tumor weight, (B) CAM intravasation and (C and D) distal metastasis in the presence and/or absence of macrophages. Data are presented as mean ± SEM of three independent experiments. MyLa: 6 embryos, n=2 independent experiments; SeAx: 6 embryos, n=2 independent experiments; ** $p < 0.01$, **** $p<0.0001$; ns: not significant (Brown-Forsythe and Welch ANOVA tests with Dunnett's T3 multiple comparison test). KO, knockout; M, macrophages; CM, Conditioned Medium.

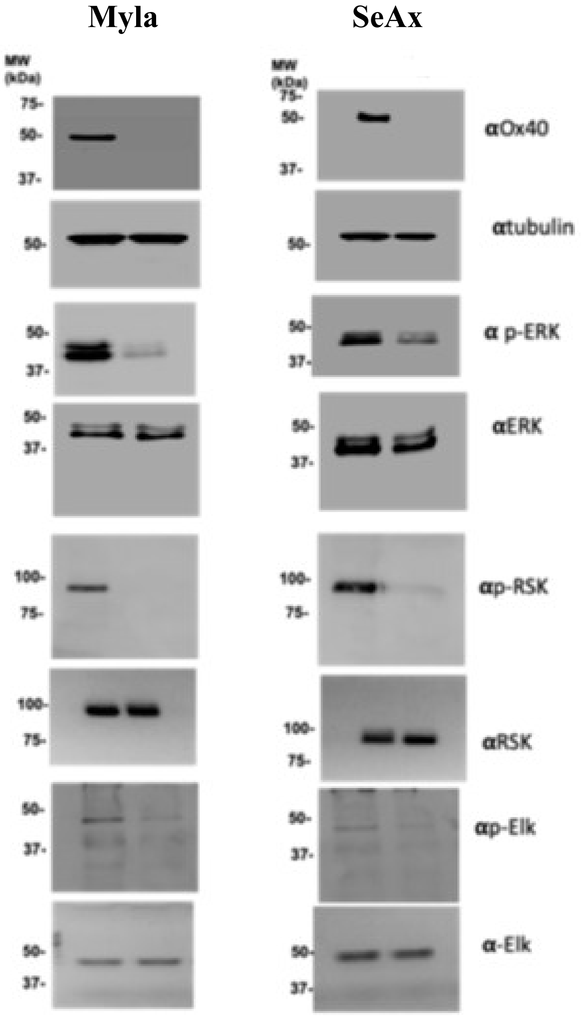

Supplementary Figure S5. Unprocessed images for Western Blot results corresponding to Figure 2

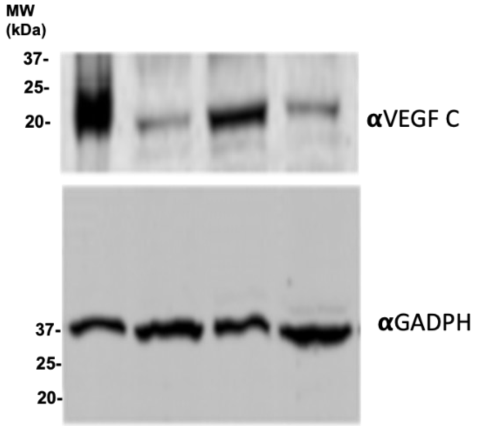

Supplementary Figure S6. Unprocessed images for Western Blot results corresponding to Figure 7
